# Alteration of medial temporal lobe metabolism related to Alzheimer’s disease and dementia with lewy bodies

**DOI:** 10.1186/s13195-024-01429-4

**Published:** 2024-04-23

**Authors:** Sungwoo Kang, Seun Jeon, Young-gun Lee, Byoung Seok Ye

**Affiliations:** 1 Department of Neurology, Yonsei University College of Medicine, 50-1 Yonsei-ro, Seodaemun-gu, Seoul, 03722 Republic of Korea; 2 Metabolism-Dementia Research Institute , Yonsei University College of Medicine, Seoul, Republic of Korea; 3 Ilsan Paik Hospital, Inje University College of Medicine, Goyang, Republic of Korea

**Keywords:** Metabolism, Alzheimer disease, Lewy body disease, Mixed dementia, Positron-emission tomography

## Abstract

**Background:**

Association of medial temporal lobe (MTL) metabolism with Alzheimer’s disease (AD) and dementia with Lewy bodies (DLB) has not been evaluated considering their mixed disease (MD).

**Methods:**

131 patients with AD, 133 with DLB, 122 with MD, and 28 normal controls (NCs) underwent neuropsychological tests, assessments for parkinsonism, cognitive fluctuation (CF), and visual hallucinations (VH), and ^18^F-fluorodeoxyglucose PET to quantify MTL metabolism in the amygdala, hippocampus, and entorhinal cortex. The effects of AD and DLB on MTL metabolism were evaluated using general linear models (GLMs). Associations between MTL metabolism, cognition, and clinical features were evaluated using GLMs or logistic regression models separately performed for the AD spectrum (NC + AD + MD), DLB spectrum (NC + DLB + MD), and disease groups (AD + DLB + MD). Covariates included age, sex, and education.

**Results:**

AD was associated with hippocampal/entorhinal hypometabolism, whereas DLB was associated with relative amygdalar/hippocampal hypermetabolism. Relative MTL hypermetabolism was associated with lower attention/visuospatial/executive scores and severe parkinsonism in both the AD and DLB spectra and disease groups. Left hippocampal/entorhinal hypometabolism was associated with lower verbal memory scores, whereas right hippocampal hypometabolism was associated with lower visual memory scores in both the AD spectrum and disease groups. Relative MTL hypermetabolism was associated with an increased risk of CF and VH in the disease group, and relative amygdalar hypermetabolism was associated with an increased risk of VH in the DLB spectrum.

**Conclusions:**

Entorhinal-hippocampal hypometabolism and relative amygdala-hippocampal hypermetabolism could be characteristics of AD- and DLB-related neurodegeneration, respectively.

**Supplementary Information:**

The online version contains supplementary material available at 10.1186/s13195-024-01429-4.

## Background

Alzheimer’s disease (AD) and Lewy body disease (LBD) are the two most common neurodegenerative and pathological causes of dementia. Although mixed pathologies of AD and LBD are common in cognitively impaired patients on autopsy; [1, 2] precise antemortem diagnosis is not easy in the real world due to the interaction of the two diseases on clinical symptoms [2–4]. For example, co-occurring AD pathologies in LBD patients lower the presentation rate of core clinical features of dementia with Lewy bodies (DLB), including visual hallucinations (VH) and cognitive fluctuation (CF) [3, 4]. Moreover, although LBD pathologies are associated with parkinsonism and more severe cognitive and behavioral deterioration in AD patients, [5] the lack of validated biomarkers has lessened clinicians’ interest in LB-related clinical symptoms.

Limbic structures are vulnerable to both AD and LBD. Neurofibrillary tangles in the entorhinal cortex and hippocampus are the cardinal neuropathological features of AD [6]. Limbic areas are also preferentially affected by Lewy bodies (LBs) in diffuse LBD, [7] and the amygdala is the most commonly affected by LB pathology in patients with AD [1, 8]. Brain metabolism on ^18^F-fluorodeoxyglucose (FDG)-PET is regarded as a useful imaging biomarker for the differential diagnosis of dementia, [9] and focusing on limbic metabolism could provide useful information for the diagnosis of DLB and AD. Previous studies have shown that the medial temporal lobe (MTL) structures are locations of hypometabolism in patients with AD, while relative preservation of the MTL is the most consistent finding in DLB patients [10–12]. However, to the best of our knowledge, the effects of AD and DLB on MTL metabolism have not been elucidated while considering these two diseases simultaneously.

In this study, we investigated the effects of AD and DLB on the metabolism of the MTL structures, including the amygdala, hippocampus, and entorhinal cortex. We also evaluated the association between MTL metabolism and cognitive dysfunction, as well as the core clinical features of DLB, including parkinsonism, CF, rapid eye movement sleep behavior disorder (RBD), and VH, separately in the AD and DLB spectra. We hypothesized that AD and DLB independently contribute to MTL metabolism with disease-specific patterns that are related, in turn, to disease-specific clinical manifestations.

## Methods

### Study participants

The study participants were 28 cognitively normal controls (NCs), 131 patients with AD, 133 patients with DLB, and 122 patients with mixed disease (MD)—having AD and DLB—recruited from the dementia clinic of Yonsei University Severance Hospital, Seoul, South Korea, from January 2015 to November 2022. NCs were recruited from a previous independent study of healthy volunteers and did not have any subjective symptoms of cognitive impairment or a history of neurological or psychiatric illnesses. They had normal cognitive function according to the Korean version of the Mini-Mental State Examination (K-MMSE) and detailed neuropsychological tests (described below). All participants underwent neuropsychological tests, Unified Parkinson’s disease motor scale (UPDRS) examination, brain magnetic resonance imaging (MRI), ^18^F-flobetaben (FBB)-PET, and FDG-PET. All patients with DLB and MD additionally underwent ^18^F-fluorinated N-3 fluoropropyl-2-betacarboxy-methoxy-3-beta-(4-iodophenyl) nortropane (FP-CIT)-PET scans. Among the 131 patients with AD, 53 (40.5%) underwent FP-CITPET scans for research purpose, which showed preserved dopamine transporter uptake in the striatum. The clinical diagnoses of AD and DLB were based on the diagnostic guidelines of the 2011 National Institute on Aging and Alzheimer’s Association [13] and the fourth consensus report of the DLB consortium published in 2017 [3]. AD-related mild cognitive impairment (MCI) was based on the National Institute on Aging-Alzheimer’s Association workgroup guidelines, [14] while MCI with Lewy bodies (MCI-LB) was based on the 2020 research criteria for MCI-LB [15]. Patients with AD who satisfied the diagnostic criteria for probable DLB were considered to have MD. The diagnoses of AD and DLB were further supported by biomarker evidence of amyloid β deposition and reduced dopamine transporter uptake in the striatum based on visual rating as well as quantitative analysis [16]. All patients with AD, all patients with MD, and 56 of 133 patients with DLB (42.1%) had significant cerebral amyloid-β deposition on FBB-PET confirmed by the quantitative analysis. Patients with DLB and MD showed abnormalities in dopamine transporter uptake on FP-CIT-PET. However, although these patients presented with cognitive impairment, parkinsonism, and DAT depletion, they were not considered to have DLB if they did not experience CF or VH. The NC participants had normal FBB-PET, FDG-PET, and FP-CIT-PET findings.

### Neuropsychological evaluation and clinical assessment

All participants completed the standardized Seoul Neuropsychological Screening Battery (SNSB), [17] which comprises tests for attention, language, visuospatial ability, memory, and frontal/executive function. Standardized z-scores were available for all tests, with scores after age- and education-level matching. We included the following tests in our analyses: the Korean version of the Boston Naming Test (K-BNT) for the language domain; the copying item of the Rey–Osterrieth Complex Figure Test (RCFT) for the visuospatial domain; the immediate recall, 20-min delayed recall, and recognition items of the RCFT and the Seoul Verbal Learning Test (SVLT) for the memory domain; and the digit span backward, semantic and phonemic Controlled Oral Word Association Test (COWAT), and Stroop Color Test for the executive domain. The scores in each cognitive domain were classified as abnormal when they were > 1 standard deviation below the normal values.

### Image acquisition and processing with MRI and PET

The participants were scanned using a Philips 3.0 T MRI scanner (Philips Achieva; Philips Medical Systems, Best, The Netherlands) with a SENSE head coil (SENSE factor = 2). T1-weighted (T1w) MRI data were obtained using a three-dimensional T1w turbo-field echo sequence with these parameters: axial acquisition matrix, 224 × 224; reconstructed matrix, 256 × 256 with 170 slices; voxel size, 0.859 × 0.859 × 1 mm^3^; field of view, 220 mm; echo time, 4.6 ms; repetition time, 9.8 ms; and flip angle, 8°.

FDG- and FBB-PET scans were performed using the Discovery 600 system (General Electric Healthcare, Milwaukee, WI, USA). The FDG-PET scans were acquired according to the following protocol: Approximately 4.1 MBq per kilogram of body weight of FDG was intravenously administered to the patients. After 60 min of uptake, the PET images were acquired for 15 min. For FBB, 300 MBq (8 mCi) was intravenously administered during the procedure. Ninety minutes after injection, images were acquired over a 20-minute session. The images were reconstructed using the ordered subset expectation maximization algorithm with four iterations and 32 subsets. A Gaussian filter with a 4-mm full width at half maximum (FWHM) kernel was applied to the reconstructed PET images, yielding a 256 × 256 matrix with 0.98-mm pixels and 0.98-mm slice thickness.

### Image processing methods

The FMRIB Software Library (FSL, http://www.fmrib.ox.ac.uk/fsl) and Advanced Normalization Tools (ANTs) were used for T1-weighted and PET image processing. Each subject’s T1-weighted images were corrected for intensity inhomogeneity, skull-stripped, and registered to the Montreal Neurological Institute (MNI) template. The tissues in the registered images were classified into white matter, gray matter (GM), or cerebrospinal fluid based on the hidden-Markov random field model and the associated expectation-maximization algorithm [18]. GM probability map obtained from this algorithm was non-linearly transformed into the MNI template. The volume of interest (VOI) in the amygdala and hippocampus was segmented based on parameterized deformable surface meshes using T1-weighted MRI and the FSL FIRST algorithm [19]. The striatal regions of interest were included in the GM class. Then, we generated a study-specific GM mask by averaging all the individual GM probability maps and binarizing the average map (> 30% GM probability), and then assigned each voxel into either background or foreground. The entorhinal cortex VOI was defined as the anterior part of the parahippocampal gyrus based on the voxel coordinates, according to the automated anatomical labeling atlas (version 3) [20], by employing the highly deformable registration algorithm implemented in the ANTs software [21]. 

FDGPET scans were coregistered to individual T1w images, spatially normalized to the MNI template, and standardized uptake value ratio (SUVR) maps of the images were generated, using the cerebellar gray matter as the reference region. The FDG-SUVR maps weresmoothed using a 6 mm FWHM Gaussian kernel and reshaped into a voxel by subject matrix within the study-specific GM mask. We transformed each data into logarithmic form and centered the data matrix by subtracting each subject mean and group mean voxel profile, resulting in a residual image, termed as the subject residual profile (SRP) that highlight deviations based on the subject and voxel group means [22]. Finally, we extracted the median FDGSRP from the VOIs.

The global amyloid burden was measured using an automated FBB-PET quantification pipeline as described elsewhere [23]. Global SUVR values for FBBPET were extracted as the cortical volume-weighted average of these cortical regions of interest: frontal, anterior/posterior cingulate, lateral parietal, and lateral temporal cortices [23]. We classified the participants as β-amyloid positive and negative applying a global FBB-SUVR cutoff value of 1.478 [24]. 

### Quality assurance for image processing

All MRI and PET images and preprocessing outcomes from the automated pipelines were visually inspected for quality assurance by three researchers (SWK, SJ, and BSY) who were blinded to the participant information.

### Statistical analysis

Statistical analyses of demographic and clinical data were performed using R statistical software (version 4.2.1). Analyses of variance and χ2 tests were performed to compare the clinical features between the disease and control groups. To compare the quantitatively measured MTL metabolism among the NC, AD, DLB, and MD groups, general linear models (GLMs) were used. To identify the independent and interaction effects of AD and DLB on MTL metabolism, we used GLMs. Interaction effects were included only if they were significant. We evaluated the effects of MTL metabolism on standardized neuropsychological z-scores and the UPDRS motor score in a combined group of patients with AD, MD, and NC (AD spectrum); a combined group of patients with DLB, MD, and NC (DLB spectrum); and a combined group of patients with AD, DLB, and MD (disease group) using GLMs. To determine the association between MTL metabolism and the risk of CF, RBD, and VH, logistic regression analyses were performed. All GLMs and logistic regression models were adjusted for age, sex, and educational level.

To explore the heterogeneity of the associations between MTL metabolism and clinical features within each disease group, sensitivity analyses were conducted in the AD, DLB, and MD group, separately.

## Results

### Demographics and clinical characteristic of the study participants

The demographic and clinical characteristics of the study participants are presented in Table 1. Compared with the NC group, all disease groups were older and had a shorter duration of education. The proportion of female patients in the DLB group was lower than in the MD group. Compared with the NC group, all disease groups had lower mean K-MMSE scores and higher clinical dementia rating (CDR). The proportion of patients with dementia in the AD group was comparable to that in the DLB group. The proportion of patients with dementia was higher in the MD group than that in the AD and DLB groups. The MD group had lower mean K-MMSE scores and higher CDR than the AD and DLB groups. All disease groups had higher mean UPDRS motor scores than the NC group, and the MD group had higher mean UPDRS motor scores than the AD group. The NC and AD groups did not exhibit any CF or VH. The DLB and MD groups had a higher proportion of CF and VH than the NC and AD groups.

**Table 1:** Comparison of demographic data among the NC, AD, DLB, MD groups

	NC (*N* = 28)	AD (*N* = 131)	DLB (*N* = 133)	MD (*N* = 122)	*P* value
Age, y	61.93 ± 7.40	74.15 ± 7.92^*^	74.44 ± 6.47^*^	73.24 ± 7.63^*^	< 0.001
Education, y	14.30 ± 4.34	10.71 ± 5.10^*^	9.80 ± 5.32^*^	9.41 ± 5.13^*^	< 0.001
Female, n (%)	16 (57.1%)	84 (64.1%)	72 (54.1%)	88 (72.1%)^‡^	0.026
K-MMSE	29.04 ± 1.07	22.44 ± 4.11^*^	22.90 ± 4.26^*^	20.87 ± 4.91^*,†,‡^	< 0.001
CDR	0.04 ± 0.13	0.59 ± 0.23^*^	0.66 ± 0.33^*^	0.78 ± 0.38^*,†,‡^	< 0.001
*Stages*					0.001
MCI, n (%)	-	70 (53.4%)	77 (57.9%)	44 (36.1%)^†,‡^	
Dementia, n (%)	-	61 (46.6%)	56 (42.1%)	78 (63.9%)^†,‡^	
UPDRS	0.96 ± 2.38	17.85 ± 10.47^*^	20.29 ± 9.70^*^	20.65 ± 10.90^*,†^	< 0.001
Cognitive fluctuation	0 (0%)	0 (0%)	107 (80.5%)^*,†^	101 (82.8%)^*,†^	< 0.001
Visual hallucination	0 (0%)	0 (0%)	22 (16.5%)^*,†^	22 (18.0%)^*,†^	< 0.001
RBD	0 (0%)	12 (9.2%)	39 (29.3%)^*,†^	28 (23.0%)^*,†^	0.008

Data are expressed as means (SD) or numbers (%). Group comparisons were performed using the chi-square test or analysis of variance, as appropriate

^*^Significantly different from NC after false discovery method correction. ^†^Significantly different from AD after false discovery method correction. ^‡^Significantly different from DLB after false discovery method correction

Abbreviations: AD, Alzheimer’s disease; CDR, clinical dementia rating; DLB, dementia with Lewy bodies; K-MMSE, Korean-mini-mental state examination; MCI, mild cognitive impairment; MD, mixed disease; NC, normal cognition; RBD, rapid eye movement sleep behavior disorder; UPDRS, Unified Parkinson’s Disease Rating Scale

### Comparison of MTL metabolism

**Fig. 1:**
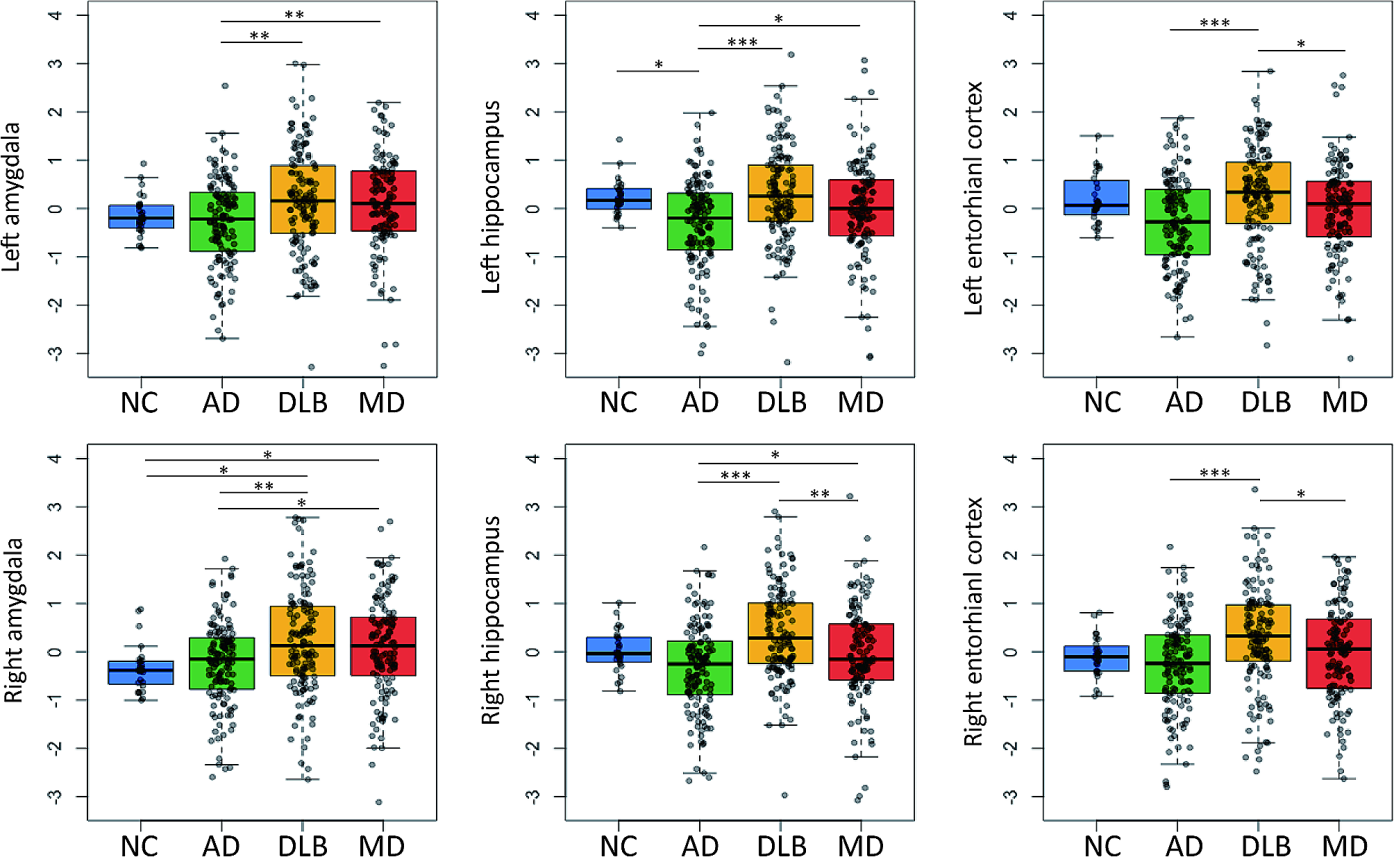
Group comparisons of metabolism in the MTL of the study groups. The data show the distribution of the z-transformed residuals in each group from the general linear models for MTL metabolism after controlling for age, sex, and education. *P*-values were obtained after correcting for multiple comparisons across 36 tests (6 MTL regions by 6 tests) with false discovery rate methods (* *P* < 0.05; ** *P* < 0.01; *** *P* < 0.001). Abbreviations: AD, Alzheimer’s disease; DLB, dementia with Lewy bodies; MD, mixed disease; MTL, medial temporal lobe; NC, normal control

Compared with the NC group, the AD group had a lower mean metabolism in the left hippocampus, whereas the DLB and MD groups had a higher mean metabolism in the right amygdala (Fig. 1). The DLB group had higher mean metabolism than the AD group in all MTL regions. The MD group had higher mean metabolism in the bilateral amygdala and hippocampus than the AD group, whereas the MD group had lower mean metabolism in the bilateral entorhinal cortex and right hippocampus than the DLB group.

### Effects of the presence of AD and DLB on MTL metabolism

**Fig. 2:**
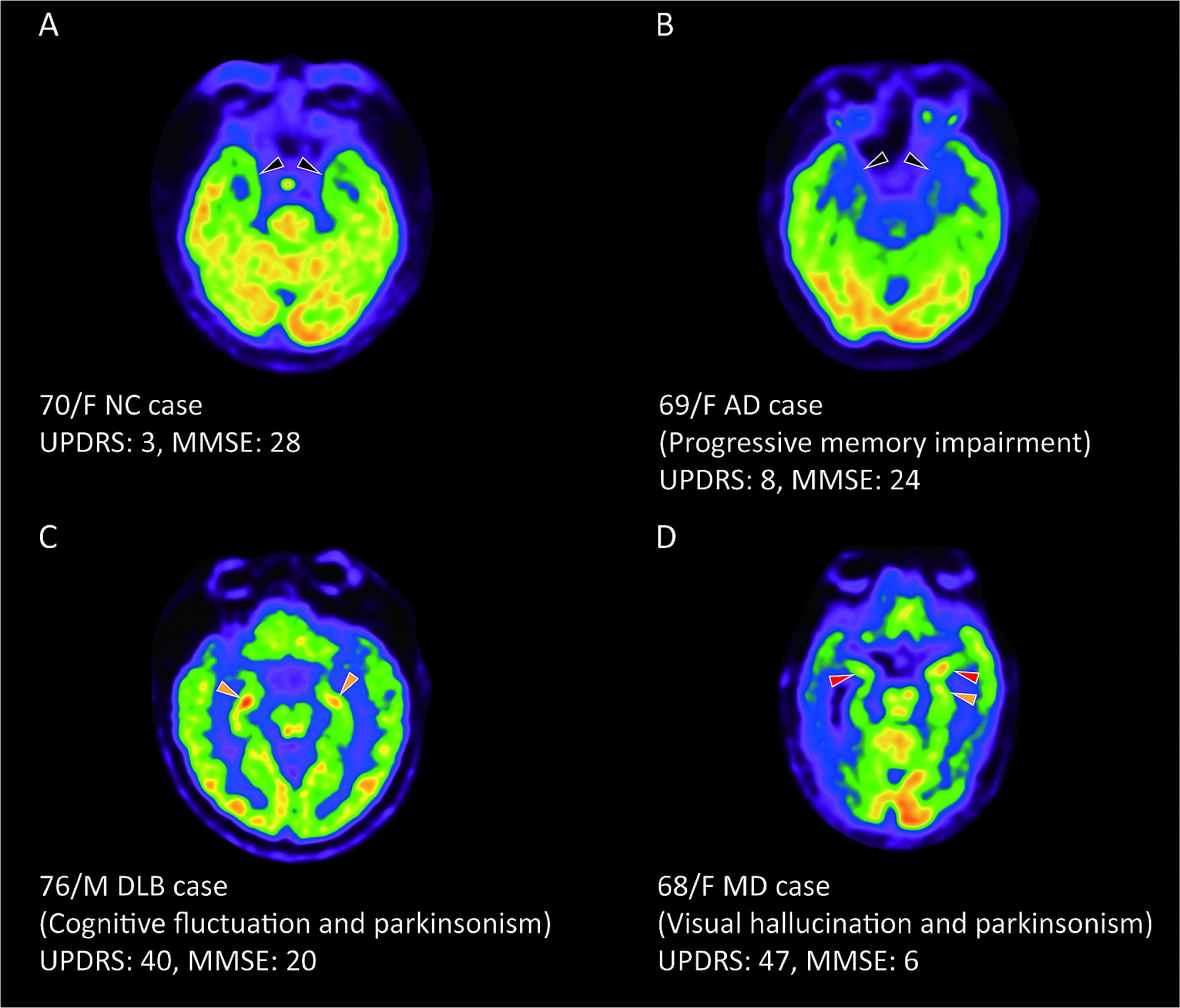
Representative examples of MTL metabolic changes in each group. **(A)** An NC participant with preserved metabolism in the bilateral entorhinal cortex (black arrows). **(B)** patient with AD with progressive memory impairment exhibiting decreased metabolism in the bilateral entorhinal cortex (black arrows). **(C)** A patient with DLB with cognitive fluctuations and parkinsonism who exhibit bilateral relative hippocampal hypermetabolism (orange arrows). **(D)** MD patient with visual hallucinations and parkinsonism who exhibits bilateral relative amygdala hypermetabolism with an emphasis on the left side (red arrows) and left relative hippocampal hypermetabolism (orange arrow). Abbreviations: AD, Alzheimer’s disease; DLB, dementia with Lewy bodies; MD, mixed disease; MTL, medial temporal lobe; MMSE, Mini-Mental State Examination; NC, normal control; UPDRS, Unified Parkinson’s Disease Rating Scale

There was no significant interaction effect between AD and DLB on the metabolism of each MTL region (data not shown); therefore, the independent effects of AD and DLB on MTL metabolism were evaluated (Table 2). AD was associated with decreased metabolism in the left hippocampus, right hippocampus, left entorhinal cortex, and right entorhinal cortex. Contrastingly, the presence of DLB was associated with increased metabolism in the left amygdala, right amygdala, left hippocampus, right hippocampus, and right entorhinal cortex. Figure 2 shows representative images of a patient with NC showing normal entorhinal metabolism (Fig. 2A), a patient with AD showing entorhinal hypometabolism (Fig. 2B), a patient with DLB showing relative hippocampal hypermetabolism (Fig. 2C), and a patient with MD showing relative amygdalar and hippocampal hypermetabolism (Fig. 2D).

**Table 2:** Independent effect of AD and DLB on MTL metabolism

	AD		DLB	
	β	*P*	β	*P*
Left amygdala	-0.02	0.688	0.19	**< 0.001**
Right amygdala	-0.02	0.689	0.20	**< 0.001**
Left hippocampus	-0.17	**0.001**	0.13	**0.015**
Right hippocampus	-0.19	**< 0.001**	0.15	**0.004**
Left entorhinal cortex	-0.17	**0.001**	0.09	0.076
Right entorhinal cortex	-0.12	**0.016**	0.14	**0.008**

Multivariable general linear models were used to investigate the independent effects of AD and DLB on MTL metabolism after controlling for age, sex, and education. Significant *P*-values are shown in boldface after false discovery rate correction for multiple statistical tests across the six MTL regions. β = standardized beta coefficient. Abbreviations: AD, Alzheimer’s disease; DLB, dementia with Lewy bodies; MTL, medial temporal lobe

### Association of MTL metabolism and cognition

Table 3 shows the univariable GLMs for neuropsychological test z-scores separately performed in the AD spectrum, DLB spectrum, and disease groups. In the AD spectrum, hypometabolism in the left hippocampus and left entorhinal cortex was associated with lower SVLT delayed recall scores, whereas hypometabolism in the right hippocampus was associated with lower RCFT delayed recall scores. Relative hypermetabolism in all MTL regions was associated with lower RCFT copy scores, and relative hypermetabolism in the bilateral amygdala was associated with lower scores on the COWAT semantic, COWAT phonemic, and Stroop color reading tests. Relative hypermetabolism in the right hippocampus was also associated with lower COWAT phonemic scores, whereas relative hypermetabolism in the right entorhinal cortex was also associated with lower scores on the COWAT phonemic and Stroop color reading tests.

**Table 3:** Association of MTL metabolism with standardized cognitive function scores

	Left amygdala		Right amygdala		Left hippocampus		Right hippocampus		Left entorhinal		Right entorhinal	
	β	*P*	β	*P*	β	*P*	β	*P*	β	*P*	β	*P*
**AD spectrum**												
Digit span backward	-0.13	0.031	-0.16	**0.007**	-0.05	0.399	-0.09	0.143	-0.12	0.062	-0.10	0.087
K-BNT	-0.12	0.044	-0.17	**0.005**	-0.07	0.252	-0.12	0.064	-0.04	0.551	-0.08	0.180
RCFT copy	-0.30	**< 0.001**	-0.30	**< 0.001**	-0.19	**0.001**	-0.16	**0.007**	-0.21	**0.001**	-0.21	**< 0.001**
SVLT immediate recall	-0.01	0.823	-0.15	**0.015**	0.16	**0.012**	-0.02	0.701	0.13	0.036	-0.04	0.487
SVLT delayed recall	0.06	0.353	-0.05	0.431	0.23	**< 0.001**	0.09	0.144	0.21	**0.001**	0.08	0.206
SVLT recognition	0.06	0.321	-0.04	0.488	0.22	**< 0.001**	0.13	0.030	0.20	**0.001**	0.12	0.052
RCFT immediate recall	-0.05	0.411	-0.05	0.391	0.10	0.097	0.10	0.094	0.12	0.066	0.06	0.320
RCFT delayed recall	-0.05	0.389	-0.05	0.443	0.13	0.029	0.15	**0.016**	0.14	0.021	0.10	0.102
RCFT recognition	-0.06	0.327	-0.01	0.878	0.15	**0.017**	0.14	0.018	0.11	0.077	0.11	0.076
COWAT semantic	-0.15	**0.013**	-0.20	**0.001**	0.00	0.936	-0.09	0.131	0.00	0.996	-0.08	0.199
COWAT phonemic	-0.22	**< 0.001**	-0.26	**< 0.001**	-0.11	0.082	-0.18	**0.004**	-0.10	0.124	-0.20	**0.002**
Stroop color reading	-0.24	**< 0.001**	-0.27	**< 0.001**	-0.12	0.053	-0.12	0.055	-0.10	0.121	-0.20	**0.001**
**DLB spectrum**												
Digit span backward	-0.24	**< 0.001**	-0.29	**< 0.001**	-0.20	**0.001**	-0.21	**< 0.001**	-0.19	**0.003**	-0.26	**< 0.001**
K-BNT	-0.15	**0.012**	-0.22	**< 0.001**	-0.09	0.150	-0.18	**0.004**	-0.05	0.446	-0.15	**0.014**
RCFT copy	-0.35	**< 0.001**	-0.35	**< 0.001**	-0.24	**< 0.001**	-0.18	**0.003**	-0.25	**< 0.001**	-0.29	**< 0.001**
SVLT immediate recall	-0.11	0.061	-0.24	**< 0.001**	0.04	0.456	-0.15	**0.012**	0.01	0.810	-0.19	**0.002**
SVLT delayed recall	-0.07	0.248	-0.15	**0.012**	0.09	0.157	-0.05	0.384	0.10	0.094	-0.04	0.527
SVLT recognition	0.00	0.972	-0.11	0.067	0.15	**0.014**	0.05	0.428	0.15	**0.015**	-0.01	0.929
RCFT immediate recall	-0.19	**0.001**	-0.15	**0.011**	-0.03	0.579	0.00	0.976	0.00	0.985	-0.02	0.751
RCFT delayed recall	-0.19	**0.002**	-0.13	**0.027**	-0.02	0.803	0.04	0.539	0.03	0.661	0.02	0.702
RCFT recognition	-0.20	**0.001**	-0.16	**0.010**	0.00	0.993	0.03	0.592	0.02	0.788	0.02	0.790
COWAT semantic	-0.24	**< 0.001**	-0.28	**< 0.001**	-0.12	0.038	-0.19	**0.001**	-0.08	0.180	-0.21	**< 0.001**
COWAT phonemic	-0.29	**< 0.001**	-0.37	**< 0.001**	-0.20	**0.001**	-0.32	**< 0.001**	-0.17	**0.007**	-0.33	**< 0.001**
Stroop color reading	-0.34	**< 0.001**	-0.37	**< 0.001**	-0.20	**0.001**	-0.21	**0.001**	-0.20	**0.001**	-0.30	**< 0.001**
**Disease group**												
Digit span backward	-0.22	**< 0.001**	-0.24	**< 0.001**	-0.18	**< 0.001**	-0.17	**0.001**	-0.21	**< 0.001**	-0.21	**< 0.001**
K-BNT	-0.12	**0.020**	-0.16	**0.002**	-0.10	0.055	-0.16	**0.002**	-0.06	0.265	-0.13	**0.016**
RCFT copy	-0.30	**< 0.001**	-0.29	**< 0.001**	-0.23	**< 0.001**	-0.17	**< 0.001**	-0.25	**< 0.001**	-0.24	**< 0.001**
SVLT immediate recall	-0.05	0.323	-0.15	**0.002**	0.06	0.195	-0.08	0.113	0.04	0.375	-0.11	**0.023**
SVLT delayed recall	0.05	0.293	-0.01	0.892	0.15	**0.002**	0.05	0.290	0.17	**< 0.001**	0.08	0.109
SVLT recognition	0.09	0.055	0.02	0.744	0.19	**< 0.001**	0.13	**0.010**	0.20	**< 0.001**	0.11	**0.027**
RCFT immediate recall	-0.07	0.132	0.00	0.996	0.01	0.770	0.09	0.058	0.05	0.322	0.08	0.091
RCFT delayed recall	-0.06	0.201	0.02	0.688	0.05	0.257	0.14	**0.004**	0.09	0.054	0.13	**0.006**
RCFT recognition	-0.08	0.093	-0.01	0.784	0.08	0.114	0.12	**0.013**	0.08	0.124	0.11	0.035
COWAT semantic	-0.20	**< 0.001**	-0.21	**< 0.001**	-0.11	**0.024**	-0.16	**0.001**	-0.07	0.147	-0.14	**0.007**
COWAT phonemic	-0.28	**< 0.001**	-0.30	**< 0.001**	-0.24	**< 0.001**	-0.30	**< 0.001**	-0.22	**< 0.001**	-0.29	**< 0.001**
Stroop color reading	-0.28	**< 0.001**	-0.26	**< 0.001**	-0.20	**< 0.001**	-0.17	**0.001**	-0.19	**< 0.001**	-0.24	**< 0.001**

Data represent the results of univariable general linear models for neuropsychological test z-scores using MTL metabolism as a predictor, after controlling for age, sex, and education. Significant *p*-values are shown in boldface after false discovery rate correction for multiple statistical testing across 72 regression analyses (six predictors by 12 cognitive tests). Analyses were performed separately on the AD spectrum (NC + AD + MD), DLB spectrum (NC + DLB + MD), and in the disease group (AD + DLB + MD). β = standardized beta coefficient. Abbreviations: AD, Alzheimer’s disease; COWAT, Controlled Oral Word Association Test; DLB, dementia with Lewy bodies; K-BNT, Korean version of the Boston Naming Test; MD, mixed disease; MTL, medial temporal lobe; NC, normal control; RCFT, Rey–Osterrieth Complex figure Test; SVLT, Seoul Verbal Learning Test

In the DLB spectrum, relative hypermetabolism in all MTL regions was associated with lower scores on the digit span backward, RCFT copy, COWAT phonemic, and Stroop color reading tests. Relative hypermetabolism in the bilateral amygdala was also associated with lower scores on the K-BNT, COWAT semantics, immediate recall, delayed recall, and recognition items of the RCFT. Relative hypermetabolism in the right amygdala was further associated with lower scores on the immediate and delayed recall items of the SVLT, whereas relative hypermetabolism in the right hippocampus and entorhinal cortex was also associated with lower scores on the K-BNT, COWAT semantic, and immediate recall items of the SVLT. In contrast to other MTL regions, hypometabolism in the left hippocampus and left entorhinal cortex was associated with lower SVLT recognition scores.

In the disease group, hypometabolism in the left hippocampus and left entorhinal cortex was associated with lower SVLT delayed recall and recognition scores. Hypometabolism in the right hippocampus was associated with lower RCFT delayed recall and recognition scores, while hypometabolism in the right entorhinal cortex was associated with lower SVLT recognition and RCFT delayed recall scores. Contrastingly, relative hypermetabolism in all MTL regions was associated with lower scores on the digit span backward, RCFT copy, phonemic COWAT, and Stroop color reading tests. Relative hypermetabolism in the bilateral amygdala was additionally associated with lower K-BNT and COWAT semantic scores, and relative hypermetabolism in the right amygdala was also associated with lower SVLT immediate recall scores. Relative hypermetabolism in the left hippocampus was also associated with lower COWAT semantic scores, and relative hypermetabolism in the right hippocampus was further associated with lower K-BNT and COWAT semantic scores. Relative hypermetabolism in the right entorhinal cortex was also associated with lower K-BNT, SVLT immediate recall, and COWAT semantic scores.

### Association of MTL metabolism and parkinsonism

Relative hypermetabolism in all the MTL regions was associated with higher UPDRS motor scores in the AD and DLB spectra, and disease groups (Table 4). Among the MTL regions, the right amygdala had the largest effect size for the AD and DLB spectra, whereas the left amygdala had the largest effect size for the disease group.

**Table 4:** Association of MTL metabolism with UPDRS motor score

	UPDRS	
	β	*P*
**AD spectrum**		
Left amygdala	0.181	**0.001**
Right amygdala	0.187	**0.001**
Left hippocampus	0.148	**0.010**
Right hippocampus	0.130	**0.023**
Left entorhinal cortex	0.123	**0.036**
Right entorhinal cortex	0.158	**0.006**
**DLB spectrum**		
Left amygdala	0.216	**< 0.001**
Right amygdala	0.227	**< 0.001**
Left hippocampus	0.162	**0.004**
Right hippocampus	0.146	**0.009**
Left entorhinal cortex	0.131	**0.022**
Right entorhinal cortex	0.178	**0.002**
**Disease group**		
Left amygdala	0.191	**< 0.001**
Right amygdala	0.174	**0.001**
Left hippocampus	0.179	**< 0.001**
Right hippocampus	0.155	**0.002**
Left entorhinal cortex	0.163	**0.002**
Right entorhinal cortex	0.161	**0.002**

The data are the results of univariable general linear models for the UPDRS motor score using MTL metabolism as a predictor after controlling for age, sex, and education. Significant *P*-values are shown in boldface after false discovery rate correction for multiple statistical tests across the six MTL regions. Analyses were performed separately on the AD spectrum (NC + AD + MD), DLB spectrum (NC + DLB + MD), and in the disease group (AD + DLB + MD). β = standardized beta coefficient. Abbreviations: AD, Alzheimer’s disease; DLB, dementia with Lewy bodies; MD, mixed disease; MTL, medial temporal lobe; NC, normal control; UPDRS, Unified Parkinson’s Disease Rating Scale

### Association of MTL metabolism and DLB core features including CF, RBD, and VH

The results of the univariable logistic regression analyses for DLB core features, including CF, RBD, and VH, are presented in Table 5. On the AD spectrum, MTL metabolism was not associated with the risk of CF, RBD, or VH. In the DLB spectrum, relative hypermetabolism in the bilateral amygdala was associated with the risk of VH. In the disease group, relative hypermetabolism in all MTL regions was associated with the risk of CF and VH.

**Table 5:** Association of MTL metabolism with cognitive fluctuation, RBD, and visual hallucinations

	Cognitive fluctuation		RBD		Visual hallucination	
	Odds ratio (95% CI)	*P*	Odds ratio (95% CI)	*P*	Odds ratio (95% CI)	*P*
**AD spectrum**						
Left amygdala	1.45 (1.08 ~ 1.98)	0.015	0.82 (0.56 ~ 1.21)	0.325	1.28 (0.79 ~ 2.10)	0.315
Right amygdala	1.50 (1.10 ~ 2.07)	0.013	0.80 (0.53 ~ 1.21)	0.292	1.07 (0.63 ~ 1.83)	0.797
Left hippocampus	1.25 (0.92 ~ 1.71)	0.161	0.83 (0.57 ~ 1.24)	0.359	1.08 (0.65 ~ 1.84)	0.785
Right hippocampus	1.47 (1.06 ~ 2.08)	0.024	0.85 (0.55 ~ 1.32)	0.472	1.01 (0.58 ~ 1.76)	0.978
Left entorhinal cortex	1.10 (0.85 ~ 1.44)	0.456	0.88 (0.62 ~ 1.24)	0.454	0.98 (0.63 ~ 1.54)	0.922
Right entorhinal cortex	1.20 (0.88 ~ 1.64)	0.257	0.96 (0.63 ~ 1.45)	0.831	1.00 (0.58 ~ 1.74)	0.999
**DLB spectrum**						
Left amygdala	1.23 (0.91 ~ 1.67)	0.188	0.95 (0.7 ~ 1.28)	0.745	1.77 (1.24 ~ 2.57)	**0.002**
Right amygdala	1.34 (0.98 ~ 1.85)	0.071	0.87 (0.64 ~ 1.19)	0.396	1.71 (1.18 ~ 2.51)	**0.005**
Left hippocampus	1.01 (0.72 ~ 1.42)	0.940	1.01 (0.72 ~ 1.43)	0.942	1.61 (1.08 ~ 2.45)	0.022
Right hippocampus	1.32 (0.94 ~ 1.88)	0.114	0.95 (0.67 ~ 1.34)	0.758	1.36 (0.90 ~ 2.08)	0.147
Left entorhinal cortex	0.97 (0.73 ~ 1.28)	0.817	1.07 (0.81 ~ 1.43)	0.630	1.38 (0.99 ~ 1.95)	0.062
Right entorhinal cortex	1.18 (0.85 ~ 1.63)	0.327	1.02 (0.74 ~ 1.41)	0.912	1.59 (1.08 ~ 2.39)	0.022
**Disease group**						
Left amygdala	1.45 (1.16 ~ 1.82)	**0.001**	0.99 (0.76 ~ 1.29)	0.932	1.97 (1.39 ~ 2.85)	**< 0.001**
Right amygdala	1.45 (1.15 ~ 1.85)	**0.002**	0.94 (0.71 ~ 1.24)	0.643	1.90 (1.31 ~ 2.78)	**0.001**
Left hippocampus	1.54 (1.21 ~ 1.97)	**0.001**	1.11 (0.84 ~ 1.49)	0.468	1.92 (1.30 ~ 2.87)	**0.001**
Right hippocampus	1.76 (1.36 ~ 2.31)	**< 0.001**	1.09 (0.81 ~ 1.47)	0.579	1.62 (1.10 ~ 2.42)	**0.016**
Left entorhinal cortex	1.31 (1.07 ~ 1.61)	**0.009**	1.15 (0.89 ~ 1.48)	0.286	1.57 (1.14 ~ 2.20)	**0.007**
Right entorhinal cortex	1.42 (1.13 ~ 1.80)	**0.003**	1.11 (0.83 ~ 1.48)	0.489	1.77 (1.21 ~ 2.62)	**0.004**

Data are the results of logistic regression models on clinical features (cognitive fluctuation, RBD, and visual hallucinations) using MTL metabolism as a predictor after controlling for age, sex, and education. Significant *P*-values are shown in boldface after correcting for multiple comparisons for 18 regression analyses (6 predictors by 3 clinical features) with false discovery rate methods in each disease spectrum. Abbreviations: AD, Alzheimer’s disease; CI, confidence interval; DLB, dementia with Lewy bodies; MTL, medial temporal lobe; RBD, rapid eye movement sleep behavior disorder

### Sensitivity analysis

The associations of MTL metabolism with cognition (Supplementary Table 1) and DLB core features (Supplementary Tables 2 and 3) were analyzed in the AD, DLB, and MD group, separately. Overall results in the sensitivity analyses were very similar to the original results.

## Discussion

In this study, we evaluated the effects of AD and DLB on MTL metabolism and the association between MTL metabolism and clinical features in the spectrum of normal aging and cognitive impairment due to AD, DLB, and MD. Our major findings were as follows. First, the presence of AD was independently associated with hypometabolism in the bilateral hippocampus and entorhinal cortex, whereas the presence of DLB was associated with relative hypermetabolism in the bilateral amygdala, hippocampus, and right entorhinal cortex. Second, left hippocampal/entorhinal hypometabolism was associated with verbal memory dysfunction, whereas right hippocampal hypometabolism was associated with visual memory dysfunction in both the AD spectrum and disease groups. Third, relative MTL hypermetabolism, especially in the amygdala, was associated with attention, visuospatial, and executive dysfunction, in addition to more severe motor parkinsonism in both the AD and DLB spectra, as well as in the disease group. Fourth, relative MTL hypermetabolism was associated with an increased risk of CF and VH in the disease group, and relative amygdala hypermetabolism was associated with an increased risk of VH in the DLB spectrum. Collectively, our results suggest that entorhinal/hippocampal hypometabolism and relative amygdalar/hippocampal hypermetabolism reflect AD- and DLB-related neurodegeneration, respectively.

The first major finding of this study was that AD was independently associated with MTL hypometabolism, whereas DLB was independently associated with relative MTL hypermetabolism. The contrasting effects of these two diseases on MTL metabolism compete in the hippocampus, whereas the effect of AD prevails in the entorhinal cortex, especially on the left side, and that of DLB in the amygdala, especially on the right side. Our results are generally in line with previous studies on AD showing decreased MTL metabolism in the amygdala, [25] hippocampus, [25, 26] and entorhinal cortex [27, 28]. Studies on DLB, [29] Parkinson’s disease (PD), [30] and even patients with RBD with later clinical progression to PD or DLB, [31] reported increased metabolism in the MTL. Although there are discrepant results showing preserved hippocampal metabolism in AD patients, [32, 33] and relatively preserved rather than increased MTL metabolism in DLB patients, [10–12] prevalent mixed pathologies and competing effects of AD and DLB on MTL metabolism could explain these discrepancies. For example, if patients with AD have concomitant DLB, the hippocampal metabolism may be preserved rather than reduced. Conversely, if patients with DLB have concomitant AD, the average MTL metabolism may be preserved rather than increased.

Our second major finding was that left hippocampal/entorhinal hypometabolism was associated with verbal memory dysfunction, whereas right hippocampal hypometabolism was associated with visual memory dysfunction, in both the AD spectrum and disease groups. These results are consistent with previous unilateral MTL lesions or epilepsy studies showing that left lesions impair verbal memory and right lesions impair non-verbal [34] or visual memory [35]. Considering that memory impairment is the cognitive hallmark of AD and that the burdens of neurofibrillary tangles in the entorhinal cortex and hippocampus are associated with memory impairment in AD, [36, 37] hippocampal/entorhinal hypometabolism could be a disease monitoring or staging biomarker for AD from healthy aging to dementia, even in patients with mixed pathologies.

Our third major finding was that relative MTL hypermetabolism, especially in the amygdala, was associated with attention, visuospatial, and executive dysfunction, as well as more severe motor parkinsonism, in both the AD and DLB spectra, as well as in the disease group. Attention, visuospatial, and executive dysfunction are cognitive hallmarks of DLB, [38] and motor parkinsonism is a core clinical feature of DLB [3]. As limbic LB pathologies is related to visuospatial and executive dysfunction in patients with DLB [39] and PD, [40] our results suggest that relative MTL hypermetabolism could be a disease monitoring biomarker for DLB, reflecting the severity of LB pathologies. Although previous studies have shown that limbic hypermetabolism correlates with dopaminergic depletion in patients with DLB [41, 42] and RBD, [31] we could not evaluate the relationship between relative MTL hypermetabolism, dopamine transporter uptake, cognitive dysfunction, and motor parkinsonism because some of our patients with AD did not undergo FP-CIT-PET. Further studies are needed to elucidate these relationships.

Our last major finding was that relative MTL hypermetabolism was associated with an increased risk of CF and VH in the disease group, and relative amygdala hypermetabolism was associated with an increased risk of VH in the DLB spectrum. There are controversies regarding whether hypermetabolism in DLB represents a disinhibition or compensatory mechanism [41]. Although recruitment of brain function to compensate for functional loss may present as hypermetabolism, just as findings observed in the early disease stage of AD, [43] the compensatory processes cannot be maintained in the late disease stage once neurodegeneration has progressed to some extent. Additionally, these compensatory mechanisms should be directed towards alleviating the clinical symptoms of degenerative diseases. In this study, relative MTL hypermetabolism was associated with the worsening of cognitive dysfunction, parkinsonism, and even VH and CF, which are regarded as specific to DLB [3]. Therefore, our results support the conjecture that relative MTL hypermetabolism represents a disinhibitory process rather than a compensatory process. The candidates for explaining such disinhibitory processes include the lack of inhibitory neurotransmitter input from the brainstem nucleus to the limbic system, [44] disinhibition of the basal ganglia [45] or cerebellar pacemaker [46] that are connected to the limbic system, [47] and abnormal neuronal hyperexcitability caused by neuronal accumulation of α-synuclein [48]. Although a previous study showed that VHs are related to LB pathology within the amygdala and parahippocampus, [49] future studies are warranted to confirm the exact underlying mechanism for metabolic perturbation in MTL structures.

### Strengths and limitations

This study has several strengths. The diagnoses of AD and DLB were carefully made, satisfying the recently revised diagnostic criteria, and were supported by abnormal results on FBB- and FP-CIT-PET, respectively. To our knowledge, this is the first study to investigate the independent effects of both AD and DLB on MTL metabolism. However, this study also has several limitations. First, the underlying causes of neurodegeneration were not confirmed by pathological examinations. Second, the cross-sectional design limited the interpretation of causal and temporal relationships. Third, the single-center setting of this study may have introduced a selection bias. Fourth, there may be a reference issue in the measurement of regional metabolism from FDG-PET. Although we used the SRP methods which is similar to the global normalization method, the pons and cerebellum were more frequently used as reference regions. However, as metabolism in the pons, cerebellum, and motor cortex is increased in patients with LBD, [42] using such regions as reference regions could overestimate the degree of hypometabolism and underestimate the degree of hypermetabolism, which was significantly associated with the core clinical features of DLB in our study. Sensitivity analyses using global normalized SUVR instead of SRP showed very similar results to the original results (Supplementary Tables 4–11). In contrast, sensitivity analyses using pons-normalized SUVR successfully captured the association between MTL hypometabolism and AD-related clinical features, such as memory dysfunction, but failed to capture the association between MTL hypermetabolism and DLB-related clinical features, including parkinsonism, CF, RBD, or VH. Notably, the sensitivity analyses to determine the independent effects of AD and DLB on regional metabolism and those to determine the association between MTL metabolism and DLB-related clinical features consistently showed that the models using globally normalized MTL metabolism had lower Akaike information criteria values than those using pons-normalized MTL metabolism. Global normalization or SRP methods may have better power to accurately detect DLB-related neurodegeneration without missing AD-related neurodegeneration. Fifth, as the MTL system is closely connected to other brain regions, metabolic alterations in related structures could confound the effects of MTL metabolism. Considering that the limbic area is rarely involved in vascular lesions, [50] MTL metabolism can capture the pathological burden more precisely than other cortical or subcortical metabolic pathways, which are vulnerable to vascular pathology. Sixth, validation in an independent sample is necessary to confirm our findings. Recently, we investigated in 62 autopsy-confirmed patients with AD and/or LBD pathologies using the AD Neuroimaging Initiative database. The previous study showed that AD pathology was associated with hypometabolism in the bilateral hippocampus, entorhinal cortex, and posterior cingulate cortex regardless of LBD pathology, whereas LBD pathology was associated with relative hypermetabolism in the bilateral putamen and anterior cingulate cortex regardless of AD pathology, [51] demonstrating robustness of the results in the current study. Seventh, our results should be interpreted with caution due to the small sample size and younger age of the NC group in comparison to the disease groups. However, the sensitivity analyses of the 12 NC subjects who were not statistically different in age from the disease groups showed almost the same results as the original results (data are not shown). Further studies are needed to replicate our results using NCs with a sufficient sample size and age comparable to the disease groups.

## Conclusions

Our results suggest that there are AD- and DLB-specific patterns of MTL metabolism. MTL metabolism on FDG-PET may be a useful biomarker for the detection and monitoring of cognitive impairment due to AD and DLB.

### Electronic supplementary material

Below is the link to the electronic supplementary material.

Supplementary Material 1

## Data Availability

To replicate the procedures and results, any qualified investigator can request anonymized data after obtaining ethics clearance and approval from all authors.

## Abbreviations

AD: Alzheimer’s disease
CDR: Clinical dementia rating
CF: Cognitive fluctuation
COWAT: Controlled oral word association test
CSF: Cerebrospinal fluid
DLB: Dementia with Lewy bodies
FBB: ^18^F-flobetaben
FDG: ^18^F-fluorodeoxyglucose
GLMs: General linear models
GM: Gray matter
K-BNT: Korean version of the Boston naming test
K-MMSE: Korean version of the Mini-Mental State Examination
LBs: Lewy bodies
LBD: Lewy body disease
NCs: Normal controls
MD: Mixed disease
MRI: Magnetic resonance imaging
MTL: Medial temporal lobe
PD: Parkinson’s disease
RCFT: Rey–Osterrieth complex figure test
RBD: Rapid eye movement sleep behavior disorder
SNSB: Seoul neuropsychological screening battery
SRP: Subject residual profile
SUVR: Standardized uptake value ratio
SVLT: Seoul verbal learning test
UPDRS: Unified Parkinson’s disease rating scale
VH: Visual hallucination

## References

[1] Hamilton RL (2000). Lewy bodies in Alzheimer’s disease: a neuropathological review of 145 cases using alpha-synuclein immunohistochemistry. Brain Pathol.

[2] Irwin DJ, Grossman M, Weintraub D, Hurtig HI, Duda JE, Xie SX (2017). Neuropathological and genetic correlates of survival and dementia onset in synucleinopathies: a retrospective analysis. Lancet Neurol.

[3] McKeith IG, Boeve BF, Dickson DW, Halliday G, Taylor JP, Weintraub D (2017). Diagnosis and management of dementia with Lewy bodies: fourth consensus report of the DLB Consortium. Neurology.

[4] Coughlin DG, Hurtig HI, Irwin DJ (2020). Pathological influences on clinical heterogeneity in Lewy Body Diseases. Mov Disord.

[5] Chung EJ, Babulal GM, Monsell SE, Cairns NJ, Roe CM, Morris JC (2015). Clinical features of Alzheimer Disease with and without Lewy Bodies. JAMA Neurol.

[6] Braak H, Braak E (1995). Staging of Alzheimer’s disease-related neurofibrillary changes. Neurobiol Aging.

[7] Rezaie P, Cairns NJ, Chadwick A, Lantos PL (1996). Lewy bodies are located preferentially in limbic areas in diffuse Lewy body disease. Neurosci Lett.

[8] Nelson PT, Abner EL, Patel E, Anderson S, Wilcock DM, Kryscio RJ (2018). The Amygdala as a locus of pathologic misfolding in neurodegenerative diseases. J Neuropathol Exp Neurol.

[9] Nestor PJ, Altomare D, Festari C, Drzezga A, Rivolta J, Walker Z (2018). Clinical utility of FDG-PET for the differential diagnosis among the main forms of dementia. Eur J Nucl Med Mol Imaging.

[10] Mak E, Su L, Williams GB, O’Brien JT (2014). Neuroimaging characteristics of dementia with Lewy bodies. Alzheimers Res Ther.

[11] Pillai JA, Wu G, Tousi B, Larvie M, Léger GC, Leverenz JB (2019). Amygdala sign, a FDG-PET signature of dementia with Lewy Bodies. Parkinsonism Relat Disord.

[12] Kantarci K, Boeve BF, Przybelski SA, Lesnick TG, Chen Q, Fields J (2021). FDG PET metabolic signatures distinguishing prodromal DLB and prodromal AD. NeuroImage. Clinical.

[13] McKhann GM, Knopman DS, Chertkow H, Hyman BT, Jack CR, Kawas CH (2011). The diagnosis of dementia due to Alzheimer’s disease: recommendations from the National Institute on Aging-Alzheimer’s Association workgroups on diagnostic guidelines for Alzheimer’s disease. Alzheimers Dement.

[14] Albert MS, DeKosky ST, Dickson D, Dubois B, Feldman HH, Fox NC (2011). The diagnosis of mild cognitive impairment due to Alzheimer’s disease: recommendations from the National Institute on Aging-Alzheimer’s Association workgroups on diagnostic guidelines for Alzheimer’s disease. Alzheimers Dement.

[15] McKeith IG, Ferman TJ, Thomas AJ, Blanc F, Boeve BF, Fujishiro H (2020). Research criteria for the diagnosis of prodromal dementia with Lewy bodies. Neurology.

[16] Lee YG, Jeon S, Kang SW, Ye BS (2023). Effects of amyloid beta and dopaminergic depletion on perfusion and clinical symptoms. Alzheimers Dement.

[17] Kang Y, Na D. Seoul Neuropsychological Screening Battery (SNSB): Human Brain Research & Consulting Co. In: Incheon; 2003.

[18] Zhang Y, Brady M, Smith S (2001). Segmentation of brain MR images through a hidden Markov random field model and the expectation-maximization algorithm. IEEE Trans Med Imaging.

[19] Patenaude B, Smith SM, Kennedy DN, Jenkinson M (2011). A bayesian model of shape and appearance for subcortical brain segmentation. NeuroImage.

[20] Rolls ET, Huang CC, Lin CP, Feng J, Joliot M (2020). Automated anatomical labelling atlas 3. NeuroImage.

[21] Avants BB, Tustison NJ, Song G, Cook PA, Klein A, Gee JC (2011). A reproducible evaluation of ANTs similarity metric performance in brain image registration. NeuroImage.

[22] Moeller JR, Strother SC (1991). A Regional Covariance Approach to the analysis of functional patterns in Positron Emission Tomographic Data. J Cereb Blood Flow Metabolism.

[23] Lee YG, Jeon S, Yoo HS, Chung SJ, Lee SK, Lee PH (2018). Amyloid-beta-related and unrelated cortical thinning in dementia with Lewy bodies. Neurobiol Aging.

[24] Sabri O, Sabbagh MN, Seibyl J, Barthel H, Akatsu H, Ouchi Y (2015). Florbetaben PET imaging to detect amyloid beta plaques in Alzheimer’s disease: phase 3 study. Alzheimers Dement.

[25] Nestor PJ, Fryer TD, Smielewski P, Hodges JR (2003). Limbic hypometabolism in Alzheimer’s disease and mild cognitive impairment. Ann Neurol.

[26] Mosconi L, Tsui WH, De Santi S, Li J, Rusinek H, Convit A (2005). Reduced hippocampal metabolism in MCI and AD: automated FDG-PET image analysis. Neurology.

[27] Karow DS, McEvoy LK, Fennema-Notestine C, Hagler DJ, Jennings RG, Brewer JB (2010). Relative capability of MR imaging and FDG PET to depict changes associated with prodromal and early Alzheimer disease. Radiology.

[28] Walhovd KB, Fjell AM, Brewer J, McEvoy LK, Fennema-Notestine C, Hagler DJ (2010). Combining MR imaging, positron-emission tomography, and CSF biomarkers in the diagnosis and prognosis of Alzheimer disease. AJNR Am J Neuroradiol.

[29] Ye BS, Lee S, Yoo H, Chung SJ, Lee YH, Choi Y (2020). Distinguishing between dementia with Lewy bodies and Alzheimer’s disease using metabolic patterns. Neurobiol Aging.

[30] Huang C, Ravdin LD, Nirenberg MJ, Piboolnurak P, Severt L, Maniscalco JS (2013). Neuroimaging markers of motor and nonmotor features of Parkinson’s disease: an 18f fluorodeoxyglucose positron emission computed tomography study. Dement Geriatr Cogn Disord.

[31] Diaz-Galvan P, Miyagawa T, Przybelski SA, Lesnick TG, Senjem ML, Jack CR (2023). Brain glucose metabolism and nigrostriatal degeneration in isolated rapid eye movement sleep behaviour disorder. Brain Commun.

[32] Ishii K, Sasaki M, Yamaji S, Sakamoto S, Kitagaki H, Mori E (1998). Relatively preserved hippocampal glucose metabolism in mild Alzheimer’s disease. Dement Geriatr Cogn Disord.

[33] Ishii K, Sasaki H, Kono AK, Miyamoto N, Fukuda T, Mori E (2005). Comparison of gray matter and metabolic reduction in mild Alzheimer’s disease using FDG-PET and voxel-based morphometric MR studies. Eur J Nucl Med Mol Imaging.

[34] Milner B (1970). BMemory and the medial temporal regions of the brain,[in Biology of memory, KH Pribram and DE Broadbent.

[35] Bonelli SB, Powell RHW, Yogarajah M, Samson RS, Symms MR, Thompson PJ (2010). Imaging memory in temporal lobe epilepsy: predicting the effects of temporal lobe resection. Brain.

[36] Nagy Z, Jobst KA, Esiri MM, Morris JH, King EM, MacDonald B (1996). Hippocampal pathology reflects memory deficit and brain imaging measurements in Alzheimer’s disease: clinicopathologic correlations using three sets of pathologic diagnostic criteria. Dementia.

[37] Reitz C, Honig L, Vonsattel JP, Tang MX, Mayeux R (2009). Memory performance is related to amyloid and tau pathology in the hippocampus. J Neurol Neurosurg Psychiatry.

[38] Kang S, Yoon SH, Na HK, Lee Y-g, Jeon S, Baik K et al. Neuropsychological comparison of patients with Alzheimer’s Disease and Dementia with Lewy Bodies. J Clin Neurol. 2023;19. 10.3988/jcn.2022.0358 PMC10622731 37455503

[39] Schneider JA, Arvanitakis Z, Yu L, Boyle PA, Leurgans SE, Bennett DA (2012). Cognitive impairment, decline and fluctuations in older community-dwelling subjects with Lewy bodies. Brain.

[40] El-Nazer R, Adler CH, Beach TG, Belden CM, Artz J, Shill HA (2019). Regional neuropathology distribution and verbal fluency impairments in Parkinson’s disease. Parkinsonism Relat Disord.

[41] Huber M, Beyer L, Prix C, Schönecker S, Palleis C, Rauchmann BS (2020). Metabolic correlates of dopaminergic loss in dementia with Lewy Bodies. Mov Disord.

[42] Kang SW, Jeon S, Lee Y-g, Park M, Baik K, Jung JH (2021). Implication of metabolic and dopamine transporter PET in dementia with Lewy bodies. Sci Rep.

[43] Celone KA, Calhoun VD, Dickerson BC, Atri A, Chua EF, Miller SL (2006). Alterations in memory networks in mild cognitive impairment and Alzheimer’s disease: an independent component analysis. J Neurosci.

[44] Li S, Varga V, Sik A, Kocsis B (2005). GABAergic control of the ascending input from the median raphe nucleus to the limbic system. J Neurophysiol.

[45] Plenz D, Kital ST (1999). A basal ganglia pacemaker formed by the subthalamic nucleus and external Globus Pallidus. Nature.

[46] Mouginot D, Gähwiler BH (1995). Characterization of synaptic connections between cortex and deep nuclei of the rat cerebellum in vitro. Neuroscience.

[47] Blatt GJ, Oblak AL, Schmahmann JD, Manto M, Schmahmann JD, Rossi F, Gruol DL, Koibuchi N (2013). Cerebellar connections with limbic circuits: anatomy and functional implications. Handbook of the Cerebellum and Cerebellar disorders.

[48] Morris M, Sanchez PE, Verret L, Beagle AJ, Guo W, Dubal D (2015). Network dysfunction in alpha-synuclein transgenic mice and human Lewy body dementia. Ann Clin Transl Neurol.

[49] Harding AJ, Stimson E, Henderson JM, Halliday GM (2002). Clinical correlates of selective pathology in the amygdala of patients with Parkinson’s disease. Brain.

[50] Shaw CM, Alvord EC (1997). Jr. Neuropathology of the limbic system. Neuroimaging Clin N Am.

[51] Lee Y-g, Jeon S, Park M, Kang SW, Yoon SH, Baik K (2022). Effects of Alzheimer and Lewy Body Disease pathologies on Brain Metabolism. Ann Neurol.

