# Supplementary material for: Alteration of medial temporal lobe metabolism related to Alzheimer’s disease and dementia with lewy bodies
Source: Alzheimers Res Ther. 2024 Apr 23;16:89. doi: 10.1186/s13195-024-01429-4 (PMC11036684; doi:10.1186/s13195-024-01429-4)
Supplement: Supplementary file 1 — Supplementary Material 1 [file 13195_2024_1429_MOESM1_ESM.docx]

**Supplementary Table 1**. Association of MTL metabolism with standardized cognitive function scores.

|  | Left amygdala | | Right amygdala | | Left hippocampus | | Right hippocampus | | Left entorhinal | | Right entorhinal | |
| --- | --- | --- | --- | --- | --- | --- | --- | --- | --- | --- | --- | --- |
|  | β | P | β | P | β | P | β | P | β | P | β | P |
| **AD** |  |  |  |  |  |  |  |  |  |  |  |  |
| Digit span backward | -0.14 | 0.147 | -0.13 | 0.179 | -0.02 | 0.852 | 0.00 | 0.963 | -0.14 | 0.115 | -0.07 | 0.446 |
| K-BNT | -0.02 | 0.863 | -0.02 | 0.823 | -0.01 | 0.946 | -0.05 | 0.562 | 0.01 | 0.916 | -0.02 | 0.832 |
| RCFT copy | -0.13 | 0.166 | -0.10 | 0.261 | -0.10 | 0.280 | -0.10 | 0.257 | -0.14 | 0.119 | -0.08 | 0.386 |
| SVLT immediate recall | -0.09 | 0.324 | -0.12 | 0.151 | 0.05 | 0.602 | 0.02 | 0.851 | 0.03 | 0.686 | -0.01 | 0.927 |
| SVLT delayed recall | 0.08 | 0.336 | 0.05 | 0.584 | 0.17 | **0.045** | 0.13 | 0.125 | 0.18 | **0.030** | 0.15 | 0.059 |
| SVLT recognition | 0.15 | 0.072 | 0.11 | 0.190 | 0.22 | **0.010** | 0.19 | **0.028** | 0.22 | **0.008** | 0.25 | **0.002** |
| RCFT immediate recall | 0.06 | 0.421 | 0.13 | 0.093 | 0.09 | 0.250 | 0.16 | **0.044** | 0.11 | 0.166 | 0.14 | 0.078 |
| RCFT delayed recall | 0.06 | 0.459 | 0.13 | 0.092 | 0.14 | 0.074 | 0.19 | **0.013** | 0.15 | **0.046** | 0.18 | **0.016** |
| RCFT recognition | 0.10 | 0.267 | 0.23 | **0.010** | 0.23 | **0.008** | 0.27 | **0.002** | 0.17 | **0.044** | 0.24 | **0.004** |
| COWAT semantic | -0.04 | 0.650 | 0.00 | 0.981 | 0.07 | 0.406 | 0.02 | 0.794 | 0.05 | 0.592 | 0.10 | 0.239 |
| COWAT phonemic | -0.21 | **0.027** | -0.11 | 0.261 | -0.15 | 0.106 | -0.09 | 0.319 | -0.19 | **0.045** | -0.11 | 0.237 |
| Stroop color reading | -0.17 | 0.065 | -0.10 | 0.252 | -0.16 | 0.074 | -0.09 | 0.347 | -0.10 | 0.258 | -0.14 | 0.127 |
| **DLB** |  |  |  |  |  |  |  |  |  |  |  |  |
| Digit span backward | -0.33 | **<0.001** | -0.34 | **<0.001** | -0.26 | **0.004** | -0.18 | 0.053 | -0.22 | **0.017** | -0.31 | **0.001** |
| K-BNT | -0.09 | 0.293 | -0.14 | 0.114 | -0.01 | 0.879 | -0.12 | 0.162 | 0.01 | 0.871 | -0.11 | 0.219 |
| RCFT copy | -0.31 | **<0.001** | -0.30 | **<0.001** | -0.23 | **0.007** | -0.15 | 0.078 | -0.24 | **0.004** | -0.28 | **0.001** |
| SVLT immediate recall | -0.17 | **0.039** | -0.25 | **0.002** | -0.03 | 0.741 | -0.12 | 0.144 | -0.06 | 0.439 | -0.23 | **0.006** |
| SVLT delayed recall | -0.10 | 0.217 | -0.14 | 0.099 | -0.01 | 0.951 | -0.11 | 0.218 | 0.07 | 0.432 | -0.05 | 0.594 |
| SVLT recognition | 0.06 | 0.503 | -0.06 | 0.489 | 0.13 | 0.125 | -0.01 | 0.947 | 0.17 | 0.061 | -0.05 | 0.596 |
| RCFT immediate recall | -0.18 | **0.034** | -0.08 | 0.351 | -0.09 | 0.307 | -0.01 | 0.928 | -0.05 | 0.592 | 0.01 | 0.891 |
| RCFT delayed recall | -0.17 | 0.053 | -0.06 | 0.467 | -0.09 | 0.321 | -0.02 | 0.790 | -0.02 | 0.819 | 0.04 | 0.645 |
| RCFT recognition | -0.18 | **0.040** | -0.12 | 0.179 | -0.02 | 0.856 | 0.04 | 0.688 | 0.02 | 0.836 | 0.04 | 0.626 |
| COWAT semantic | -0.22 | **0.007** | -0.21 | **0.012** | -0.11 | 0.177 | -0.14 | 0.117 | -0.06 | 0.457 | -0.15 | 0.086 |
| COWAT phonemic | -0.34 | **<0.001** | -0.37 | **<0.001** | -0.29 | **0.001** | -0.35 | **<0.001** | -0.26 | **0.002** | -0.34 | **<0.001** |
| Stroop color reading | -0.34 | **<0.001** | -0.30 | **0.001** | -0.24 | **0.006** | -0.21 | **0.020** | -0.25 | **0.005** | -0.30 | **0.001** |
| **MD** |  |  |  |  |  |  |  |  |  |  |  |  |
| Digit span backward | -0.12 | 0.190 | -0.16 | 0.071 | -0.16 | 0.070 | -0.22 | **0.014** | -0.19 | **0.035** | -0.17 | 0.070 |
| K-BNT | -0.16 | 0.072 | -0.23 | **0.013** | -0.20 | **0.030** | -0.21 | **0.022** | -0.13 | 0.172 | -0.16 | 0.097 |
| RCFT copy | -0.33 | **<0.001** | -0.30 | **<0.001** | -0.29 | **<0.001** | -0.19 | **0.021** | -0.28 | **0.001** | -0.26 | **0.002** |
| SVLT immediate recall | 0.06 | 0.478 | -0.10 | 0.256 | 0.11 | 0.225 | -0.18 | **0.041** | 0.11 | 0.225 | -0.10 | 0.275 |
| SVLT delayed recall | 0.15 | 0.054 | 0.07 | 0.362 | 0.18 | **0.028** | -0.01 | 0.898 | 0.18 | **0.032** | 0.08 | 0.348 |
| SVLT recognition | 0.07 | 0.389 | -0.02 | 0.848 | 0.12 | 0.158 | 0.05 | 0.560 | 0.11 | 0.199 | 0.05 | 0.579 |
| RCFT immediate recall | -0.11 | 0.151 | -0.06 | 0.468 | -0.07 | 0.351 | 0.00 | 0.970 | -0.01 | 0.922 | 0.02 | 0.829 |
| RCFT delayed recall | -0.13 | 0.084 | -0.05 | 0.530 | -0.05 | 0.502 | 0.08 | 0.316 | 0.01 | 0.911 | 0.06 | 0.407 |
| RCFT recognition | -0.15 | 0.068 | -0.10 | 0.240 | -0.05 | 0.575 | 0.00 | 0.983 | -0.02 | 0.848 | 0.01 | 0.932 |
| COWAT semantic | -0.22 | **0.012** | -0.31 | **<0.001** | -0.23 | **0.010** | -0.31 | **<0.001** | -0.14 | 0.140 | -0.28 | **0.001** |
| COWAT phonemic | -0.22 | **0.020** | -0.35 | **<0.001** | -0.22 | **0.024** | -0.38 | **<0.001** | -0.13 | 0.183 | -0.34 | **<0.001** |
| Stroop color reading | -0.29 | **0.001** | -0.35 | **<0.001** | -0.24 | **0.009** | -0.23 | **0.010** | -0.21 | **0.025** | -0.28 | **0.003** |

The data represent the results of univariable general linear models for standardized neuropsychological test scores using MTL metabolism as a predictor after controlling for age, sex, and education. Analyses were performed separately for AD (N = 131), DLB (N = 133), and MD (N = 122) groups. β = standardized beta coefficient. Abbreviations: AD, Alzheimer’s disease; COWAT, Controlled Oral Word Association Test; DLB, dementia with Lewy bodies; K-BNT, Korean version of the Boston Naming Test; MD, mixed disease; NC, normal control; RCFT, Rey–Osterrieth Complex figure Test; SVLT, Seoul Verbal Learning test

**Supplementary Table 2**. Association of MTL metabolism with UPDRS motor score.

|  | UPDRS | |
| --- | --- | --- |
|  | β | P |
| **AD** |  |  |
| Left amygdala | 0.07 | 0.470 |
| Right amygdala | 0.08 | 0.413 |
| Left hippocampus | 0.06 | 0.544 |
| Right hippocampus | 0.09 | 0.321 |
| Left entorhinal cortex | 0.08 | 0.352 |
| Right entorhinal cortex | 0.10 | 0.257 |
| **DLB** |  |  |
| Left amygdala | 0.19 | **0.026** |
| Right amygdala | 0.20 | **0.025** |
| Left hippocampus | 0.12 | 0.183 |
| Right hippocampus | 0.13 | 0.145 |
| Left entorhinal cortex | 0.12 | 0.169 |
| Right entorhinal cortex | 0.13 | 0.141 |
| **MD** |  |  |
| Left amygdala | 0.23 | **0.011** |
| Right amygdala | 0.21 | **0.022** |
| Left hippocampus | 0.31 | **0.001** |
| Right hippocampus | 0.20 | **0.034** |
| Left entorhinal cortex | 0.23 | **0.015** |
| Right entorhinal cortex | 0.22 | **0.016** |

The data are the results of univariable general linear models for the UPDRS motor score using MTL metabolism as a predictor after controlling for age, sex, and education. Analyses were performed separately for AD (N = 131), DLB (N = 133), and MD (N = 122) groups. β = standardized beta coefficient. Abbreviations: AD, Alzheimer’s disease; DLB, dementia with Lewy bodies; MD, mixed disease; NC, normal control; UPDRS, Unified Parkinson’s Disease Rating Scale

**Supplementary Table 3.** Association of MTL metabolism with cognitive fluctuations, RBD, and visual hallucinations.

|  | Cognitive fluctuation | | RBD | | Visual hallucination | |
| --- | --- | --- | --- | --- | --- | --- |
|  | Odds ratio (95% CI) | P | Odds ratio (95% CI) | P | Odds ratio (95% CI) | P |
| **AD** |  |  |  |  |  |  |
| Left amygdala | - | - | 0.75 (0.35~1.63) | 0.465 | - | - |
| Right amygdala | - | - | 1.12 (0.51~2.56) | 0.779 | - | - |
| Left hippocampus | - | - | 0.79 (0.41~1.59) | 0.482 | - | - |
| Right hippocampus | - | - | 1.11 (0.49~2.48) | 0.804 | - | - |
| Left entorhinal cortex | - | - | 0.90 (0.46~1.77) | 0.758 | - | - |
| Right entorhinal cortex | - | - | 1.24 (0.58~2.81) | 0.586 | - | - |
| **DLB** |  |  |  |  |  |  |
| Left amygdala | 1.31 (0.82~2.12) | 0.259 | 1.15 (0.76~1.75) | 0.519 | 2.90 (1.65~5.58) | **0.001** |
| Right amygdala | 1.12 (0.69~1.82) | 0.636 | 1.08 (0.71~1.66) | 0.724 | 2.89 (1.64~5.54) | **0.001** |
| Left hippocampus | 1.24 (0.72~2.14) | 0.442 | 1.33 (0.82~2.22) | 0.259 | 3.00 (1.58~6.27) | **0.002** |
| Right hippocampus | 1.14 (0.66~1.96) | 0.629 | 1.20 (0.74~2.00) | 0.475 | 2.02 (1.09~3.99) | **0.033** |
| Left entorhinal cortex | 1.10 (0.71~1.70) | 0.654 | 1.36 (0.91~2.10) | 0.150 | 2.24 (1.33~4.13) | **0.005** |
| Right entorhinal cortex | 1.19 (0.72~1.95) | 0.490 | 1.20 (0.77~1.91) | 0.434 | 2.63 (1.43~5.38) | **0.004** |
| **MD** |  |  |  |  |  |  |
| Left amygdala | 0.87 (0.52~1.45) | 0.594 | 0.71 (0.44~1.13) | 0.154 | 1.01 (0.62~1.65) | 0.960 |
| Right amygdala | 1.02 (0.59~1.76) | 0.940 | 0.57 (0.34~0.94) | **0.031** | 0.88 (0.52~1.48) | 0.631 |
| Left hippocampus | 0.83 (0.46~1.43) | 0.502 | 0.83 (0.50~1.36) | 0.453 | 0.87 (0.50~1.51) | 0.612 |
| Right hippocampus | 1.43 (0.80~2.59) | 0.227 | 0.73 (0.43~1.24) | 0.241 | 0.83 (0.47~1.48) | 0.523 |
| Left entorhinal cortex | 0.82 (0.50~1.33) | 0.439 | 0.85 (0.56~1.29) | 0.442 | 0.88 (0.56~1.38) | 0.567 |
| Right entorhinal cortex | 0.87 (0.48~1.51) | 0.617 | 0.78 (0.47~1.30) | 0.342 | 0.88 (0.50~1.54) | 0.662 |

The data are the results of logistic regression models of clinical features (cognitive fluctuations, RBD, and visual hallucinations) using MTL metabolism as a predictor after controlling for age, sex, and education. Abbreviations: AD, Alzheimer’s disease; CI, confidence interval; DLB, dementia with Lewy bodies; RBD, rapid eye movement sleep behavior disorder

**Supplementary Table 4**. Independent effect of AD and DLB on global or pons normalized MTL metabolism.

|  | AD |  | DLB |  |  |
| --- | --- | --- | --- | --- | --- |
|  | β | P | β | P | AIC |
| *Global normalized* |  |  |  |  |  |
| Left amygdala | -0.01 | 0.850 | 0.20 | **< 0.001** | -1064.79 |
| Right amygdala | -0.02 | 0.723 | 0.21 | **< 0.001** | -1091.66 |
| Left hippocampus | -0.14 | **0.006** | 0.14 | **0.007** | -1090.91 |
| Right hippocampus | -0.18 | **0.001** | 0.16 | **0.002** | -1121.63 |
| Left entorhinal cortex | -0.10 | 0.045 | 0.16 | **0.003** | -1043.22 |
| Right entorhinal cortex | -0.10 | 0.051 | 0.16 | **0.002** | -1109.73 |
| *Pons normalized* |  |  |  |  |  |
| Left amygdala | -0.24 | **< 0.001** | 0.01 | 0.846 | -998.46 |
| Right amygdala | -0.22 | **< 0.001** | 0.001 | 0.977 | -1009.49 |
| Left hippocampus | -0.32 | **< 0.001** | -0.07 | 0.147 | -1013.27 |
| Right hippocampus | -0.33 | **< 0.001** | -0.05 | 0.307 | -995.35 |
| Left entorhinal cortex | -0.32 | **< 0.001** | -0.06 | 0.239 | -1043.18 |
| Right entorhinal cortex | -0.28 | **< 0.001** | -0.07 | 0.176 | -1044.58 |

Multivariable general linear models were used to investigate the independent effects of AD and DLB on MTL metabolism after controlling for age, sex, and education. Significant P-values are shown in boldface after false discovery rate correction for multiple statistical tests across the six MTL regions. β = standardized beta coefficient. Abbreviations: AD, Alzheimer’s disease; AIC, Akaike information criterion; DLB, dementia with Lewy bodies

**Supplementary Table 5**. Association of global or pons-normalized MTL metabolism with standardized cognitive function scores in AD spectrum.

|  | Left amygdala | | | Right amygdala | | | Left hippocampus | | | Right hippocampus | | | Left entorhinal | | | Right entorhinal | | |
| --- | --- | --- | --- | --- | --- | --- | --- | --- | --- | --- | --- | --- | --- | --- | --- | --- | --- | --- |
|  | β | P | AIC | β | P | AIC | β | P | AIC | β | P | AIC | β | P | AIC | β | P | AIC |
| **Global normalized** |  |  |  |  |  |  |  |  |  |  |  |  |  |  |  |  |  |  |
| Digit span backward | -0.15 | **0.013** | 865.91 | -0.18 | **0.002** | 862.67 | -0.09 | 0.148 | 870.04 | -0.13 | 0.028 | 867.28 | -0.15 | **0.017** | 866.37 | -0.16 | **0.011** | 865.50 |
| K-BNT | -0.16 | **0.011** | 954.83 | -0.20 | **0.001** | 950.45 | -0.13 | 0.042 | 957.14 | -0.18 | **0.004** | 952.74 | -0.13 | 0.039 | 957.00 | -0.13 | 0.034 | 956.75 |
| RCFT copy | -0.33 | **<0.001** | 1242.04 | -0.31 | **<0.001** | 1244.83 | -0.25 | **<0.001** | 1256.85 | -0.24 | **<0.001** | 1258.12 | -0.31 | **<0.001** | 1246.95 | -0.26 | **<0.001** | 1254.23 |
| SVLT immediate recall | -0.04 | 0.534 | 869.48 | -0.17 | **0.007** | 862.52 | 0.10 | 0.123 | 867.45 | -0.07 | 0.245 | 868.50 | 0.05 | 0.404 | 869.17 | -0.08 | 0.206 | 868.25 |
| SVLT delayed recall | 0.03 | 0.624 | 891.78 | -0.06 | 0.354 | 891.15 | 0.16 | **0.011** | 885.37 | 0.04 | 0.525 | 891.61 | 0.11 | 0.087 | 889.03 | 0.03 | 0.636 | 891.79 |
| SVLT recognition | 0.03 | 0.620 | 1008.44 | -0.06 | 0.345 | 1007.78 | 0.17 | **0.005** | 1000.73 | 0.10 | 0.111 | 1006.09 | 0.11 | 0.076 | 1005.48 | 0.07 | 0.234 | 1007.24 |
| RCFT immediate recall | -0.08 | 0.192 | 779.15 | -0.07 | 0.220 | 779.36 | 0.05 | 0.402 | 780.17 | 0.06 | 0.366 | 780.06 | 0.02 | 0.756 | 780.79 | 0.03 | 0.608 | 780.62 |
| RCFT delayed recall | -0.09 | 0.152 | 805.02 | -0.07 | 0.248 | 805.75 | 0.08 | 0.180 | 805.27 | 0.10 | 0.099 | 804.34 | 0.04 | 0.550 | 806.75 | 0.06 | 0.330 | 806.15 |
| RCFT recognition | -0.08 | 0.208 | 966.80 | -0.02 | 0.783 | 968.34 | 0.10 | 0.119 | 965.94 | 0.11 | 0.088 | 965.45 | 0.04 | 0.558 | 968.07 | 0.06 | 0.338 | 967.48 |
| COWAT semantic | -0.18 | **0.003** | 691.35 | -0.22 | **<0.001** | 687.56 | -0.06 | 0.308 | 699.48 | -0.15 | **0.019** | 694.90 | -0.12 | 0.061 | 696.94 | -0.13 | 0.038 | 696.16 |
| COWAT phonemic | -0.23 | **<0.001** | 772.07 | -0.28 | **<0.001** | 764.82 | -0.14 | 0.027 | 781.26 | -0.24 | **<0.001** | 771.80 | -0.18 | **0.006** | 778.50 | -0.23 | **<0.001** | 772.98 |
| Stroop color reading | -0.27 | **<0.001** | 988.61 | -0.30 | **<0.001** | 982.75 | -0.18 | **0.005** | 999.93 | -0.19 | **0.002** | 998.58 | -0.19 | **0.003** | 998.72 | -0.24 | **<0.001** | 993.20 |
| **Pons normalized** |  |  |  |  |  |  |  |  |  |  |  |  |  |  |  |  |  |  |
| Digit span backward | 0.04 | 0.546 | 871.81 | 0.04 | 0.471 | 871.65 | 0.11 | 0.074 | 868.92 | 0.08 | 0.186 | 870.40 | 0.08 | 0.227 | 870.69 | 0.07 | 0.283 | 871.00 |
| K-BNT | 0.13 | 0.042 | 957.14 | 0.12 | 0.055 | 957.58 | 0.16 | **0.010** | 954.59 | 0.13 | **0.035** | 956.82 | 0.18 | **0.005** | 953.47 | 0.19 | **0.003** | 952.54 |
| RCFT copy | 0.02 | 0.700 | 1273.98 | 0.05 | 0.413 | 1273.45 | 0.12 | 0.045 | 1270.04 | 0.15 | **0.014** | 1267.91 | 0.07 | 0.295 | 1273.01 | 0.12 | 0.045 | 1270.03 |
| SVLT immediate recall | 0.27 | **<0.001** | 850.75 | 0.15 | **0.021** | 864.47 | 0.37 | **<0.001** | 834.09 | 0.22 | **<0.001** | 857.06 | 0.38 | **<0.001** | 833.43 | 0.23 | **<0.001** | 856.20 |
| SVLT delayed recall | 0.33 | **<0.001** | 863.62 | 0.24 | **<0.001** | 877.38 | 0.40 | **<0.001** | 849.15 | 0.29 | **<0.001** | 870.26 | 0.40 | **<0.001** | 850.43 | 0.31 | **<0.001** | 868.27 |
| SVLT recognition | 0.28 | **<0.001** | 987.41 | 0.20 | **0.002** | 998.76 | 0.37 | **<0.001** | 971.43 | 0.31 | **<0.001** | 983.24 | 0.37 | **<0.001** | 972.09 | 0.33 | **<0.001** | 980.41 |
| RCFT immediate recall | 0.29 | **<0.001** | 758.24 | 0.27 | **<0.001** | 761.48 | 0.37 | **<0.001** | 743.32 | 0.35 | **<0.001** | 745.48 | 0.38 | **<0.001** | 742.17 | 0.36 | **<0.001** | 745.60 |
| RCFT delayed recall | 0.33 | **<0.001** | 777.37 | 0.33 | **<0.001** | 777.50 | 0.43 | **<0.001** | 753.92 | 0.42 | **<0.001** | 755.35 | 0.44 | **<0.001** | 754.55 | 0.42 | **<0.001** | 757.19 |
| RCFT recognition | 0.19 | **0.002** | 958.16 | 0.24 | **<0.001** | 952.33 | 0.32 | **<0.001** | 941.28 | 0.31 | **<0.001** | 941.76 | 0.31 | **<0.001** | 942.94 | 0.33 | **<0.001** | 939.62 |
| COWAT semantic | 0.23 | **<0.001** | 686.80 | 0.22 | **0.001** | 688.32 | 0.31 | **<0.001** | 675.00 | 0.26 | **<0.001** | 683.32 | 0.31 | **<0.001** | 675.46 | 0.28 | **<0.001** | 680.96 |
| COWAT phonemic | 0.07 | 0.262 | 784.95 | 0.07 | 0.286 | 785.08 | 0.17 | **0.008** | 779.09 | 0.11 | 0.076 | 783.03 | 0.15 | **0.022** | 780.90 | 0.11 | 0.082 | 783.16 |
| Stroop color reading | 0.06 | 0.305 | 1006.97 | 0.07 | 0.303 | 1006.96 | 0.15 | **0.018** | 1002.32 | 0.15 | **0.018** | 1002.31 | 0.18 | **0.005** | 999.97 | 0.13 | 0.049 | 1004.08 |

The data represent the results of univariable general linear models for standardized neuropsychological test scores using MTL metabolism as a predictor, after controlling for age, sex, and education. Significant P-values are shown in boldface after false discovery rate correction for multiple statistical testing across 72 regression analyses (six predictors by 12 cognitive tests). β = standardized beta coefficient. Abbreviations: AD, Alzheimer’s disease; AIC, Akaike information criterion; COWAT, Controlled Oral Word Association Test; K-BNT, Korean version of the Boston Naming Test; RCFT, Rey–Osterrieth Complex figure Test; SVLT, Seoul Verbal Learning Test

**Supplementary Table 6**. Association of global or pons-normalized MTL metabolism with standardized cognitive function scores in DLB spectrum.

|  | Left amygdala | | | Right amygdala | | | Left hippocampus | | | | Right hippocampus | | | Left entorhinal | | | | Right entorhinal | | |
| --- | --- | --- | --- | --- | --- | --- | --- | --- | --- | --- | --- | --- | --- | --- | --- | --- | --- | --- | --- | --- |
|  | β | P | AIC | β | P | AIC | β | P | AIC | β | | P | AIC | β | P | AIC | β | | P | AIC |
| **Global normalized** |  |  |  |  |  |  |  |  |  |  | |  |  |  |  |  |  | |  |  |
| Digit span backward | -0.26 | **<0.001** | 905.10 | -0.30 | **<0.001** | 898.96 | -0.22 | **<0.001** | 910.85 | -0.23 | | **<0.001** | 909.52 | -0.23 | **<0.001** | 909.62 | -0.31 | | **<0.001** | 897.77 |
| K-BNT | -0.18 | **0.002** | 1039.91 | -0.24 | **<0.001** | 1033.59 | -0.12 | 0.050 | 1045.41 | -0.20 | | **0.001** | 1038.13 | -0.13 | 0.034 | 1044.72 | -0.21 | | **0.001** | 1038.13 |
| RCFT copy | -0.36 | **<0.001** | 1343.68 | -0.35 | **<0.001** | 1347.66 | -0.26 | **<0.001** | 1363.71 | -0.22 | | **<0.001** | 1370.08 | -0.34 | **<0.001** | 1350.26 | -0.33 | | **<0.001** | 1354.06 |
| SVLT immediate recall | -0.13 | 0.033 | 879.25 | -0.24 | **<0.001** | 866.94 | -0.004 | 0.943 | 883.90 | -0.19 | | **0.002** | 874.18 | -0.07 | 0.260 | 882.61 | -0.22 | | **<0.001** | 870.83 |
| SVLT delayed recall | -0.08 | 0.163 | 899.03 | -0.15 | **0.013** | 894.75 | 0.03 | 0.588 | 900.71 | -0.09 | | 0.127 | 898.63 | -0.005 | 0.939 | 901.01 | -0.09 | | 0.170 | 899.09 |
| SVLT recognition | -0.02 | 0.734 | 1025.33 | -0.12 | 0.051 | 1021.57 | 0.10 | 0.089 | 1022.50 | 0.01 | | 0.869 | 1025.42 | 0.05 | 0.429 | 1024.81 | -0.06 | | 0.372 | 1024.63 |
| RCFT immediate recall | -0.20 | **0.001** | 786.03 | -0.16 | **0.006** | 790.24 | -0.06 | 0.319 | 796.79 | -0.03 | | 0.590 | 797.51 | -0.10 | 0.113 | 795.25 | -0.06 | | 0.350 | 796.91 |
| RCFT delayed recall | -0.20 | **0.001** | 806.77 | -0.15 | **0.015** | 811.98 | -0.04 | 0.492 | 817.58 | 0.01 | | 0.838 | 818.02 | -0.07 | 0.288 | 816.91 | -0.02 | | 0.735 | 817.95 |
| RCFT recognition | -0.20 | **0.001** | 977.97 | -0.15 | **0.014** | 982.90 | -0.04 | 0.468 | 988.51 | 0.001 | | 0.993 | 989.05 | -0.06 | 0.350 | 988.15 | -0.02 | | 0.795 | 988.98 |
| COWAT semantic | -0.26 | **<0.001** | 728.96 | -0.28 | **<0.001** | 725.54 | -0.16 | **0.006** | 741.65 | -0.22 | | **<0.001** | 736.26 | -0.19 | **0.002** | 739.81 | -0.26 | | **<0.001** | 730.21 |
| COWAT phonemic | -0.30 | **<0.001** | 784.35 | -0.38 | **<0.001** | 769.23 | -0.22 | **<0.001** | 796.81 | -0.34 | | **<0.001** | 777.54 | -0.24 | **<0.001** | 795.23 | -0.36 | | **<0.001** | 776.29 |
| Stroop color reading | -0.36 | **<0.001** | 967.13 | -0.38 | **<0.001** | 963.27 | -0.24 | **<0.001** | 988.96 | -0.26 | | **<0.001** | 986.72 | -0.31 | **<0.001** | 979.69 | -0.34 | | **<0.001** | 973.74 |
| **Pons normalized** |  |  |  |  |  |  |  |  |  |  | |  |  |  |  |  |  | |  |  |
| Digit span backward | -0.07 | 0.293 | 923.40 | -0.08 | 0.216 | 922.97 | 0.01 | 0.849 | 924.49 | 0.003 | | 0.968 | 924.53 | -0.003 | 0.959 | 924.53 | -0.05 | | 0.420 | 923.87 |
| K-BNT | 0.09 | 0.137 | 1047.06 | 0.08 | 0.230 | 1047.85 | 0.19 | **0.004** | 1040.71 | 0.12 | | 0.062 | 1045.76 | 0.17 | **0.007** | 1041.82 | 0.14 | | **0.023** | 1044.07 |
| RCFT copy | 0.01 | 0.881 | 1384.83 | 0.02 | 0.680 | 1384.68 | 0.16 | **0.011** | 1378.29 | 0.18 | | **0.002** | 1375.49 | 0.07 | 0.235 | 1383.41 | 0.12 | | 0.046 | 1380.77 |
| SVLT immediate recall | 0.10 | 0.091 | 880.99 | -0.002 | 0.973 | 883.90 | 0.26 | **<0.001** | 866.46 | 0.08 | | 0.172 | 882.00 | 0.21 | **0.001** | 872.26 | 0.07 | | 0.285 | 882.74 |
| SVLT delayed recall | 0.21 | **0.001** | 888.82 | 0.16 | **0.009** | 894.01 | 0.34 | **<0.001** | 870.67 | 0.20 | | **0.001** | 890.34 | 0.33 | **<0.001** | 873.23 | 0.24 | | **<0.001** | 885.97 |
| SVLT recognition | 0.18 | **0.003** | 1016.47 | 0.11 | 0.070 | 1022.09 | 0.32 | **<0.001** | 999.19 | 0.21 | | **<0.001** | 1013.07 | 0.31 | **<0.001** | 1000.91 | 0.21 | | **0.001** | 1014.11 |
| RCFT immediate recall | 0.14 | **0.021** | 792.37 | 0.18 | **0.003** | 788.64 | 0.29 | **<0.001** | 775.95 | 0.28 | | **<0.001** | 776.21 | 0.27 | **<0.001** | 778.39 | 0.32 | | **<0.001** | 769.93 |
| RCFT delayed recall | 0.17 | **0.005** | 809.94 | 0.23 | **<0.001** | 803.34 | 0.34 | **<0.001** | 788.03 | 0.34 | | **<0.001** | 785.13 | 0.33 | **<0.001** | 790.29 | 0.37 | | **<0.001** | 780.56 |
| RCFT recognition | 0.09 | 0.143 | 986.85 | 0.17 | **0.005** | 980.85 | 0.26 | **<0.001** | 971.59 | 0.27 | | **<0.001** | 968.09 | 0.26 | **<0.001** | 971.35 | 0.31 | | **<0.001** | 963.79 |
| COWAT semantic | 0.05 | 0.447 | 748.80 | 0.04 | 0.467 | 748.85 | 0.18 | **0.003** | 740.56 | 0.12 | | 0.039 | 745.06 | 0.18 | **0.004** | 740.85 | 0.11 | | 0.068 | 746.00 |
| COWAT phonemic | -0.05 | 0.440 | 809.78 | -0.09 | 0.141 | 808.17 | 0.09 | 0.163 | 808.40 | -0.01 | | 0.883 | 810.37 | 0.06 | 0.384 | 809.61 | -0.01 | | 0.870 | 810.36 |
| Stroop color reading | -0.03 | 0.683 | 1005.15 | -0.01 | 0.812 | 1005.26 | 0.16 | **0.016** | 999.35 | 0.12 | | 0.055 | 1001.56 | 0.11 | 0.097 | 1002.51 | 0.09 | | 0.168 | 1003.38 |

The data represent the results of univariable general linear models for standardized neuropsychological test scores using MTL metabolism as a predictor, after controlling for age, sex, and education. Significant p-values are shown in boldface after false discovery rate correction for multiple statistical testing across 72 regression analyses (six predictors by 12 cognitive tests). β = standardized beta coefficient. Abbreviations: AIC, Akaike information criterion; COWAT, Controlled Oral Word Association Test; DLB, dementia with Lewy bodies; K-BNT, Korean version of the Boston Naming Test; RCFT, Rey–Osterrieth Complex figure Test; SVLT, Seoul Verbal Learning Test

**Supplementary Table 7**. Association of global or pons-normalized MTL metabolism with standardized cognitive function scores in disease group.

|  | Left amygdala | | | Right amygdala | | | Left hippocampus | | | Right hippocampus | | | Left entorhinal | | | Right entorhinal | | |
| --- | --- | --- | --- | --- | --- | --- | --- | --- | --- | --- | --- | --- | --- | --- | --- | --- | --- | --- |
|  | β | P | AIC | β | P | AIC | β | P | AIC | β | P | AIC | β | P | AIC | β | P | AIC |
| **Global normalized** |  |  |  |  |  |  |  |  |  |  |  |  |  |  |  |  |  |  |
| Digit span backward | -0.23 | **<0.001** | 1160.19 | -0.25 | **<0.001** | 1156.10 | -0.20 | **<0.001** | 1165.26 | -0.19 | **<0.001** | 1166.21 | -0.23 | **<0.001** | 1161.37 | -0.25 | **<0.001** | 1156.72 |
| K-BNT | -0.14 | **0.005** | 1346.47 | -0.18 | **<0.001** | 1341.69 | -0.12 | **0.019** | 1348.77 | -0.19 | **<0.001** | 1340.99 | -0.12 | **0.019** | 1348.78 | -0.17 | **0.001** | 1343.54 |
| RCFT copy | -0.31 | **<0.001** | 1718.83 | -0.29 | **<0.001** | 1724.91 | -0.25 | **<0.001** | 1734.57 | -0.21 | **<0.001** | 1742.93 | -0.31 | **<0.001** | 1721.37 | -0.28 | **<0.001** | 1730.14 |
| SVLT immediate recall | -0.06 | 0.231 | 1020.65 | -0.16 | **0.001** | 1011.43 | 0.03 | 0.500 | 1021.64 | -0.11 | **0.029** | 1017.24 | -0.01 | 0.898 | 1022.09 | -0.13 | **0.008** | 1014.97 |
| SVLT delayed recall | 0.03 | 0.475 | 972.77 | -0.01 | 0.793 | 973.22 | 0.11 | **0.023** | 968.05 | 0.02 | 0.722 | 973.16 | 0.11 | **0.025** | 968.17 | 0.05 | 0.349 | 972.40 |
| SVLT recognition | 0.08 | 0.126 | 1279.20 | 0.003 | 0.945 | 1281.57 | 0.16 | **0.001** | 1270.60 | 0.11 | **0.031** | 1276.86 | 0.15 | **0.004** | 1272.97 | 0.08 | 0.115 | 1279.05 |
| RCFT immediate recall | -0.08 | 0.083 | 896.17 | -0.02 | 0.693 | 899.06 | -0.002 | 0.964 | 899.22 | 0.06 | 0.175 | 897.36 | 0.002 | 0.973 | 899.22 | 0.07 | 0.169 | 897.30 |
| RCFT delayed recall | -0.08 | 0.105 | 933.87 | -0.001 | 0.979 | 936.54 | 0.03 | 0.463 | 935.99 | 0.11 | **0.015** | 930.53 | 0.04 | 0.412 | 935.85 | 0.10 | **0.029** | 931.72 |
| RCFT recognition | -0.08 | 0.091 | 1302.68 | -0.02 | 0.765 | 1305.49 | 0.04 | 0.423 | 1304.93 | 0.10 | 0.054 | 1301.80 | 0.03 | 0.524 | 1305.17 | 0.07 | 0.160 | 1303.57 |
| COWAT semantic | -0.21 | **<0.001** | 885.66 | -0.22 | **<0.001** | 885.08 | -0.14 | **0.004** | 896.05 | -0.19 | **<0.001** | 890.29 | -0.16 | **0.002** | 894.74 | -0.18 | **<0.001** | 891.08 |
| COWAT phonemic | -0.28 | **<0.001** | 985.49 | -0.32 | **<0.001** | 976.87 | -0.25 | **<0.001** | 994.17 | -0.33 | **<0.001** | 975.26 | -0.26 | **<0.001** | 992.56 | -0.32 | **<0.001** | 978.51 |
| Stroop color reading | -0.29 | **<0.001** | 1276.53 | -0.28 | **<0.001** | 1278.35 | -0.23 | **<0.001** | 1290.30 | -0.22 | **<0.001** | 1293.00 | -0.25 | **<0.001** | 1287.90 | -0.27 | **<0.001** | 1282.60 |
| **Pons normalized** |  |  |  |  |  |  |  |  |  |  |  |  |  |  |  |  |  |  |
| Digit span backward | -0.12 | 0.023 | 1175.13 | -0.09 | 0.084 | 1177.34 | -0.04 | 0.400 | 1179.66 | -0.03 | 0.538 | 1179.99 | -0.08 | 0.137 | 1178.13 | -0.08 | 0.131 | 1178.07 |
| K-BNT | 0.02 | 0.679 | 1354.20 | 0.03 | 0.594 | 1354.08 | 0.07 | 0.174 | 1352.49 | 0.03 | 0.577 | 1354.06 | 0.07 | 0.192 | 1352.64 | 0.06 | 0.267 | 1353.12 |
| RCFT copy | -0.08 | 0.110 | 1759.86 | -0.04 | 0.410 | 1761.76 | 0.03 | 0.547 | 1762.08 | 0.08 | 0.108 | 1759.82 | -0.04 | 0.460 | 1761.90 | 0.03 | 0.563 | 1762.11 |
| SVLT immediate recall | 0.05 | 0.351 | 1021.22 | -0.05 | 0.342 | 1021.19 | 0.16 | **0.001** | 1011.53 | 0.03 | 0.486 | 1021.61 | 0.14 | **0.005** | 1013.96 | -0.01 | 0.873 | 1022.08 |
| SVLT delayed recall | 0.16 | **0.001** | 962.64 | 0.12 | **0.013** | 967.05 | 0.23 | **<0.001** | 951.48 | 0.13 | **0.007** | 965.88 | 0.25 | **<0.001** | 947.24 | 0.15 | **0.002** | 963.61 |
| SVLT recognition | 0.13 | **0.008** | 1274.33 | 0.08 | 0.118 | 1279.09 | 0.21 | **<0.001** | 1263.05 | 0.16 | **0.001** | 1270.95 | 0.23 | **<0.001** | 1259.13 | 0.15 | **0.003** | 1272.34 |
| RCFT immediate recall | 0.09 | 0.069 | 895.86 | 0.14 | **0.003** | 890.16 | 0.16 | **0.001** | 887.72 | 0.20 | **<0.001** | 880.57 | 0.18 | **<0.001** | 885.27 | 0.23 | **<0.001** | 874.77 |
| RCFT delayed recall | 0.13 | **0.005** | 928.43 | 0.20 | **<0.001** | 917.74 | 0.23 | **<0.001** | 912.56 | 0.28 | **<0.001** | 900.76 | 0.25 | **<0.001** | 908.52 | 0.29 | **<0.001** | 896.23 |
| RCFT recognition | 0.09 | 0.095 | 1302.75 | 0.17 | **0.001** | 1293.69 | 0.21 | **<0.001** | 1288.39 | 0.24 | **<0.001** | 1281.79 | 0.21 | **<0.001** | 1287.43 | 0.26 | **<0.001** | 1278.20 |
| COWAT semantic | 0.02 | 0.707 | 904.30 | 0.05 | 0.340 | 903.52 | 0.12 | **0.019** | 898.88 | 0.08 | 0.106 | 901.79 | 0.12 | **0.014** | 898.38 | 0.09 | 0.072 | 901.17 |
| COWAT phonemic | -0.12 | **0.022** | 1011.86 | -0.10 | 0.054 | 1013.45 | -0.02 | 0.671 | 1017.03 | -0.07 | 0.169 | 1015.30 | -0.04 | 0.450 | 1016.64 | -0.06 | 0.252 | 1015.89 |
| Stroop color reading | -0.11 | 0.036 | 1306.69 | -0.06 | 0.283 | 1309.98 | 0.00 | 0.981 | 1311.15 | 0.02 | 0.749 | 1311.04 | 0.01 | 0.834 | 1311.10 | -0.02 | 0.732 | 1311.03 |

The data represent the results of univariable general linear models for standardized neuropsychological test scores using MTL metabolism as a predictor, after controlling for age, sex, and education. Significant p-values are shown in boldface after false discovery rate correction for multiple statistical testing across 72 regression analyses (six predictors by 12 cognitive tests). β = standardized beta coefficient. Abbreviations: AIC, Akaike information criterion; COWAT, Controlled Oral Word Association Test; K-BNT, Korean version of the Boston Naming Test; RCFT, Rey–Osterrieth Complex figure Test; SVLT, Seoul Verbal Learning Test

**Supplementary Table 8**. Association of global or pons-normalized MTL metabolism with UPDRS motor score.

|  | *Global normalized* | | | *Pons normalized* | | |
| --- | --- | --- | --- | --- | --- | --- |
|  | UPDRS | | | UPDRS | | |
|  | β | P | AIC | β | P | AIC |
| **AD spectrum** |  |  |  |  |  |  |
| Left amygdala | 0.20 | **<0.001** | 2010.87 | -0.02 | 0.780 | 2023.26 |
| Right amygdala | 0.19 | **0.001** | 2012.07 | -0.01 | 0.803 | 2023.27 |
| Left hippocampus | 0.16 | **0.004** | 2014.96 | -0.03 | 0.617 | 2023.08 |
| Right hippocampus | 0.14 | **0.013** | 2017.04 | -0.05 | 0.363 | 2022.49 |
| Left entorhinal cortex | 0.17 | **0.003** | 2014.27 | -0.04 | 0.493 | 2022.86 |
| Right entorhinal cortex | 0.17 | **0.003** | 2014.30 | -0.05 | 0.426 | 2022.69 |
| **DLB spectrum** |  |  |  |  |  |  |
| Left amygdala | 0.22 | **<0.001** | 2094.81 | -0.05 | 0.384 | 2111.06 |
| Right amygdala | 0.22 | **<0.001** | 2095.64 | -0.05 | 0.400 | 2111.11 |
| Left hippocampus | 0.17 | **0.002** | 2101.83 | -0.09 | 0.139 | 2109.60 |
| Right hippocampus | 0.15 | **0.008** | 2104.67 | -0.11 | 0.062 | 2108.26 |
| Left entorhinal cortex | 0.21 | **<0.001** | 2098.15 | -0.06 | 0.314 | 2110.79 |
| Right entorhinal cortex | 0.20 | **<0.001** | 2098.64 | -0.11 | 0.048 | 2107.86 |
| **Disease group** |  |  |  |  |  |  |
| Left amygdala | 0.20 | **<0.001** | 2763.23 | 0.04 | 0.406 | 2778.50 |
| Right amygdala | 0.17 | **0.001** | 2767.37 | 0.01 | 0.786 | 2779.13 |
| Left hippocampus | 0.18 | **<0.001** | 2766.18 | 0.04 | 0.469 | 2778.67 |
| Right hippocampus | 0.16 | **0.002** | 2769.75 | 0.00 | 0.978 | 2779.20 |
| Left entorhinal cortex | 0.20 | **<0.001** | 2764.05 | 0.04 | 0.446 | 2778.61 |
| Right entorhinal cortex | 0.18 | **0.001** | 2767.08 | -0.01 | 0.813 | 2779.15 |

The data are the results of univariable general linear models for the UPDRS motor score using MTL metabolism as a predictor after controlling for age, sex, and education. Significant P-values are shown in boldface after false discovery rate correction for multiple statistical tests across the six MTL regions. Analyses were performed separately on the AD spectrum (NC+AD+MD), DLB spectrum (NC+DLB+MD), and in the disease group (AD+DLB+MD). β = standardized beta coefficient. Abbreviations: AD, Alzheimer’s disease; AIC, Akaike information criterion; DLB, dementia with Lewy bodies; MD, mixed disease; NC, normal control; UPDRS, Unified Parkinson’s Disease Rating Scale

**Supplementary Table 9.** Association of global or pons-normalized MTL metabolism with cognitive fluctuations, RBD, and visual hallucinations in AD spectrum.

|  | Cognitive fluctuation | | | RBD | | | Visual hallucination | | |
| --- | --- | --- | --- | --- | --- | --- | --- | --- | --- |
|  | Odds ratio (95% CI) | P | AIC | Odds ratio (95% CI) | P | AIC | Odds ratio (95% CI) | P | AIC |
| *Global normalized* |  |  |  |  |  |  |  |  |  |
| Left amygdala | 1.78 (1.18~2.74) | 0.007 | 357.85 | 0.73 (0.42~1.26) | 0.270 | 236.27 | 1.59 (0.83~3.05) | 0.153 | 155.81 |
| Right amygdala | 1.86 (1.22~2.90) | 0.005 | 357.24 | 0.69 (0.38~1.21) | 0.200 | 235.84 | 1.40 (0.69~2.82) | 0.351 | 156.95 |
| Left hippocampus | 1.47 (0.98~2.25) | 0.064 | 362.05 | 0.78 (0.45~1.34) | 0.367 | 236.70 | 1.33 (0.67~2.63) | 0.417 | 157.16 |
| Right hippocampus | 1.76 (1.14~2.77) | 0.013 | 359.06 | 0.83 (0.46~1.46) | 0.517 | 237.10 | 1.18 (0.57~2.41) | 0.641 | 157.60 |
| Left entorhinal cortex | 1.38 (0.94~2.04) | 0.100 | 362.85 | 0.75 (0.44~1.27) | 0.296 | 236.41 | 1.2 0(0.62~2.27) | 0.586 | 157.52 |
| Right entorhinal cortex | 1.37 (0.89~2.12) | 0.150 | 363.50 | 0.87 (0.49~1.55) | 0.647 | 237.31 | 1.18 (0.55~2.51) | 0.669 | 157.63 |
| *Pons normalized* |  |  |  |  |  |  |  |  |  |
| Left amygdala | 1.01 (0.72~1.42) | 0.964 | 365.59 | 0.89 (0.56~1.42) | 0.627 | 237.28 | 0.63 (0.34~1.13) | 0.127 | 155.45 |
| Right amygdala | 0.97 (0.68~1.37) | 0.851 | 365.56 | 0.90 (0.56~1.44) | 0.664 | 237.33 | 0.53 (0.28~0.97) | 0.044 | 153.61 |
| Left hippocampus | 0.83 (0.59~1.15) | 0.263 | 364.33 | 0.99 (0.63~1.54) | 0.953 | 237.52 | 0.51 (0.27~0.94) | 0.035 | 153.07 |
| Right hippocampus | 0.92 (0.66~1.28) | 0.620 | 365.35 | 0.98 (0.62~1.54) | 0.916 | 237.51 | 0.51 (0.27~0.92) | 0.029 | 152.80 |
| Left entorhinal cortex | 0.81 (0.57~1.15) | 0.243 | 364.22 | 0.81 (0.50~1.30) | 0.383 | 236.76 | 0.43 (0.22~0.80) | 0.009 | 150.60 |
| Right entorhinal cortex | 0.79 (0.55~1.13) | 0.193 | 363.89 | 0.94 (0.58~1.53) | 0.799 | 237.46 | 0.41 (0.20~0.79) | 0.010 | 150.63 |

Data are the results of logistic regression models on clinical features (cognitive fluctuation, RBD, and visual hallucinations) using MTL metabolism as a predictor after controlling for age, sex, and education. Significant P-values are shown in boldface after correcting for multiple comparisons for 18 regression analyses (6 predictors by 3 clinical features) with false discovery rate methods in each disease spectrum. Abbreviations: AD, Alzheimer’s disease; CI, confidence interval; DLB, dementia with Lewy bodies; RBD, rapid eye movement sleep behavior disorder

**Supplementary Table 10.** Association of global or pons-normalized MTL metabolism with cognitive fluctuation, RBD, and visual hallucinations in DLB spectrum.

|  | Cognitive fluctuation | | | RBD | | | Visual hallucination | | |
| --- | --- | --- | --- | --- | --- | --- | --- | --- | --- |
|  | Odds ratio (95% CI) | P | AIC | Odds ratio (95% CI) | P | AIC | Odds ratio (95% CI) | P | AIC |
| *Global normalized* |  |  |  |  |  |  |  |  |  |
| Left amygdala | 1.33 (0.89~2.03) | 0.170 | 309.84 | 0.90 (0.60~1.34) | 0.625 | 313.02 | 2.32 (1.47~3.77) | **<0.001** | 235.45 |
| Right amygdala | 1.45 (0.96~2.23) | 0.081 | 308.64 | 0.77 (0.51~1.16) | 0.215 | 311.70 | 2.26 (1.41~3.71) | **0.001** | 237.30 |
| Left hippocampus | 1.18 (0.77~1.83) | 0.445 | 311.18 | 1.02 (0.67~1.56) | 0.918 | 313.25 | 2.16 (1.32~3.60) | **0.003** | 239.35 |
| Right hippocampus | 1.44 (0.93~2.23) | 0.102 | 309.04 | 0.97 (0.63~1.48) | 0.891 | 313.24 | 1.68 (1.02~2.79) | 0.044 | 244.59 |
| Left entorhinal cortex | 1.09 (0.74~1.63) | 0.659 | 311.57 | 0.97 (0.65~1.44) | 0.886 | 313.24 | 1.79 (1.14~2.84) | **0.012** | 242.35 |
| Right entorhinal cortex | 1.27 (0.83~1.96) | 0.266 | 310.52 | 0.95 (0.62~1.45) | 0.803 | 313.20 | 2.13 (1.28~3.63) | **0.004** | 240.09 |
| *Pons normalized* |  |  |  |  |  |  |  |  |  |
| Left amygdala | 0.68 (0.45~1.00) | 0.052 | 307.87 | 1.14 (0.78~1.67) | 0.505 | 312.82 | 0.84 (0.55~1.30) | 0.443 | 248.13 |
| Right amygdala | 0.74 (0.49~1.11) | 0.150 | 309.65 | 1.07 (0.73~1.57) | 0.740 | 313.15 | 0.81 (0.52~1.25) | 0.340 | 247.80 |
| Left hippocampus | 0.57 (0.38~0.84) | 0.005 | 303.63 | 1.31 (0.89~1.93) | 0.171 | 311.37 | 0.67 (0.43~1.05) | 0.082 | 245.62 |
| Right hippocampus | 0.72 (0.49~1.06) | 0.101 | 308.98 | 1.19 (0.83~1.73) | 0.351 | 312.38 | 0.62 (0.41~0.92) | 0.020 | 243.23 |
| Left entorhinal cortex | 0.59 (0.39~0.87) | 0.009 | 304.70 | 1.20 (0.81~1.78) | 0.366 | 312.44 | 0.62 (0.39~0.98) | 0.041 | 244.44 |
| Right entorhinal cortex | 0.69 (0.46~1.03) | 0.074 | 308.53 | 1.16 (0.78~1.73) | 0.458 | 312.71 | 0.61 (0.38~0.98) | 0.045 | 244.57 |

Data are the results of logistic regression models on clinical features (cognitive fluctuation, RBD, and visual hallucinations) using MTL metabolism as a predictor after controlling for age, sex, and education. Significant P-values are shown in boldface after correcting for multiple comparisons for 18 regression analyses (6 predictors by 3 clinical features) with false discovery rate methods in each disease spectrum. Abbreviations: AD, Alzheimer’s disease; CI, confidence interval; DLB, dementia with Lewy bodies; RBD, rapid eye movement sleep behavior disorder

**Supplementary Table 11.** Association of global or pons-normalized MTL metabolism with cognitive fluctuation, RBD, and visual hallucinations in disease group.

|  | Cognitive fluctuation | | | RBD | | | Visual hallucination | | |
| --- | --- | --- | --- | --- | --- | --- | --- | --- | --- |
|  | Odds ratio (95% CI) | P | AIC | Odds ratio (95% CI) | P | AIC | Odds ratio (95% CI) | P | AIC |
| *Global normalized* |  |  |  |  |  |  |  |  |  |
| Left amygdala | 1.67 (1.23~2.30) | **0.001** | 527.49 | 0.97 (0.67~1.39) | 0.856 | 391.15 | 2.70 (1.72~4.38) | **<0.001** | 263.28 |
| Right amygdala | 1.70 (1.24~2.35) | **0.001** | 527.52 | 0.87 (0.60~1.26) | 0.467 | 390.65 | 2.65 (1.65~4.33) | **<0.001** | 265.70 |
| Left hippocampus | 1.82 (1.33~2.54) | **<0.001** | 524.28 | 1.14 (0.79~1.65) | 0.491 | 390.71 | 2.59 (1.61~4.26) | **<0.001** | 266.57 |
| Right hippocampus | 2.06 (1.48~2.91) | **<0.001** | 519.22 | 1.13 (0.78~1.64) | 0.524 | 390.78 | 2.07 (1.28~3.37) | **0.003** | 273.27 |
| Left entorhinal cortex | 1.55 (1.16~2.10) | **0.003** | 529.77 | 1.07 (0.75~1.51) | 0.72 | 391.06 | 2.12 (1.36~3.32) | **0.001** | 270.98 |
| Right entorhinal cortex | 1.60 (1.17~2.21) | **0.004** | 529.99 | 1.07 (0.73~1.57) | 0.732 | 391.07 | 2.45 (1.49~4.12) | **0.001** | 269.41 |
| *Pons normalized* |  |  |  |  |  |  |  |  |  |
| Left amygdala | 1.36 (1.03~1.80) | 0.034 | 534.07 | 1.35 (0.95~1.93) | 0.100 | 388.43 | 1.07 (0.70~1.64) | 0.772 | 282.07 |
| Right amygdala | 1.26 (0.96~1.68) | 0.100 | 535.94 | 1.24 (0.87~1.77) | 0.234 | 389.75 | 0.97 (0.63~1.49) | 0.883 | 282.13 |
| Left hippocampus | 1.26 (0.95~1.67) | 0.113 | 536.12 | 1.60 (1.12~2.31) | 0.012 | 384.61 | 0.86 (0.56~1.33) | 0.498 | 281.69 |
| Right hippocampus | 1.35 (1.03~1.79) | 0.031 | 533.91 | 1.47 (1.04~2.1) | 0.032 | 386.44 | 0.75 (0.50~1.13) | 0.165 | 280.25 |
| Left entorhinal cortex | 1.25 (0.94~1.68) | 0.130 | 536.35 | 1.47 (1.02~2.14) | 0.041 | 386.93 | 0.80 (0.50~1.25) | 0.321 | 281.16 |
| Right entorhinal cortex | 1.22 (0.91~1.63) | 0.185 | 536.89 | 1.43 (0.99~2.08) | 0.057 | 387.51 | 0.76 (0.48~1.20) | 0.239 | 280.75 |

The data are the results of logistic regression models on clinical features (cognitive fluctuation, RBD, and visual hallucinations) using MTL metabolism as a predictor after controlling for age, sex, and education. Significant P-values are shown in boldface after correcting for multiple comparisons for 18 regression analyses (6 predictors by 3 clinical features) with false discovery rate methods in each disease spectrum. Abbreviations: AD, Alzheimer’s disease; CI, confidence interval; DLB, dementia with Lewy bodies; RBD, rapid eye movement sleep behavior disorder
